# Natural variation of piRNA expression affects immunity to transposable elements

**DOI:** 10.1371/journal.pgen.1006731

**Published:** 2017-04-27

**Authors:** Sergei Ryazansky, Elizaveta Radion, Anastasia Mironova, Natalia Akulenko, Yuri Abramov, Valeriya Morgunova, Maria Y. Kordyukova, Ivan Olovnikov, Alla Kalmykova

**Affiliations:** Institute of Molecular Genetics, Russian Academy of Sciences, Moscow, Russia; University of Utah School of Medicine, UNITED STATES

## Abstract

In the *Drosophila* germline, transposable elements (TEs) are silenced by PIWI-interacting RNA (piRNA) that originate from distinct genomic regions termed piRNA clusters and are processed by PIWI-subfamily Argonaute proteins. Here, we explore the variation in the ability to restrain an alien TE in different *Drosophila* strains. The *I*-element is a retrotransposon involved in the phenomenon of I-R hybrid dysgenesis in *Drosophila melanogaster*. Genomes of R strains do not contain active *I*-elements, but harbour remnants of ancestral *I*-related elements. The permissivity to *I*-element activity of R females, called reactivity, varies considerably in natural R populations, indicating the existence of a strong natural polymorphism in defense systems targeting transposons. To reveal the nature of such polymorphisms, we compared ovarian small RNAs between R strains with low and high reactivity and show that reactivity negatively correlates with the ancestral *I*-element-specific piRNA content. Analysis of piRNA clusters containing remnants of *I*-elements shows increased expression of the piRNA precursors and enrichment by the Heterochromatin Protein 1 homolog, Rhino, in weak R strains, which is in accordance with stronger piRNA expression by these regions. To explore the nature of the differences in piRNA production, we focused on two R strains, weak and strong, and showed that the efficiency of maternal inheritance of piRNAs as well as the *I*-element copy number are very similar in both strains. At the same time, germline and somatic uni-strand piRNA clusters generate more piRNAs in strains with low reactivity, suggesting the relationship between the efficiency of primary piRNA production and variable response to TE invasions. The strength of adaptive genome defense is likely driven by naturally occurring polymorphisms in the rapidly evolving piRNA pathway proteins. We hypothesize that hyper-efficient piRNA production is contributing to elimination of a telomeric retrotransposon *HeT-A*, which we have observed in one particular transposon-resistant R strain.

## Author summary

Transposon activity in the germline is suppressed by the PIWI-interacting RNA (piRNA) pathway. The resistance of natural *Drosophila* strains to transposon invasion varies considerably, but the nature of this variability is unknown. We discovered that natural variation in the efficiency of primary piRNA production in the germline causes dramatic differences in the susceptibility to expansion of a newly invaded transposon. A high level of piRNA production in the germline is achieved by increased expression of piRNA precursors. In one of the most transposon-resistant strains, increased content of primary piRNA is observed in both the germline and ovarian somatic cells. We suggest that polymorphisms in piRNA pathway factors are responsible for increased piRNA production. piRNA pathway proteins have been shown to be evolving rapidly under selective pressure. Our data are the first to describe a phenotype that might be caused by this kind of polymorphism. We also demonstrate a likely explanation as to why an overly active piRNA pathway can cause more harm than good in *Drosophila*: Highly efficient piRNA processing leads to elimination of domesticated telomeric retrotransposons essential for telomere elongation, an effect which has been observed in a natural strain that is extremely resistant to transposon invasion.

## Introduction

The main function of the PIWI-interacting RNA (piRNA) system in *Drosophila* is suppression of transposon activity in the germline. piRNAs are processed from long transcripts, piRNA-precursors, encoded by distinct genomic regions enriched in TE remnants, termed piRNA clusters [1]. piRNAs recognize complementary targets, exerting RNA silencing at post-transcriptional and transcriptional levels [2]. In some cases, piRNAs cause transformation of a target locus into a novel piRNA cluster that amplifies piRNA response [3,4,5]. piRNAs generated in ovarian nurse cells are transmitted into the oocyte to launch the processing of piRNA cluster transcripts in the germline of the progeny through an epigenetic mechanism [6]. Thus, the maternal pool of piRNAs silences those TEs in the progeny that are present in the maternal genome. Invasion of alien TEs through paternal inheritance triggers a sterility syndrome, termed hybrid dysgenesis. This occurs due to the absence of maternally transmitted piRNAs complementary to the TE inherited with the paternal genome. However, through several generations, TE silencing is established as a result of the generation of corresponding piRNAs by paternal TE copies or by *de novo* TE insertions within endogenous piRNA clusters [6,7].

The phenomenon of I-R hybrid dysgenesis accompanied by female sterility is caused by mobilization of the non-LTR (long terminal repeat) retrotransposon *I*-element in crosses between reactive (R) and inducer (I) *D*. *melanogaster* strains [8]. R strains are natural *D*. *melanogaster* strains collected before the 1950s, the genomes of which do not contain active *I*-elements but do contain remnants of ancestral *I*-related elements from previous invasions [9,10]. The active *I*-element has reinvaded natural populations of *D*. *melanogaster* in the middle of 20th century. All modern natural populations of *D*. *melanogaster* carry active *I*-elements and are therefore inducers [11]. In a cross of an R female with an I male, hybrid dysgenesis is observed in the F1 progeny because of the very low amount of maternally inherited piRNAs derived from the ancient *I*-element fragments [6]. The permissivity to *I*-element activity is measured as a percentage of non-hatching embryos laid by dysgenic females; this ratio is called reactivity [10]. Previously, it was observed that transgenes that contain a transcribed fragment of an *I*-element cause suppression of dysgenic syndrome in I-R crosses [12]. We have shown that these transgenes produce *I*-specific small RNAs, which reduce the reactivity of the transgenic lines [4]. Moreover, *I*-transgenes inserted in euchromatin become *de novo* piRNA clusters. In this case, suppression of hybrid dysgenesis was achieved by artificially introduced transgenic constructs acting as an additional source of transposon-specific piRNAs. Another well-known genetic system of hybrid dysgenesis caused by the paternal contribution of a *P*-element is also at least partially related to the lack of maternally deposited *P*-element-specific piRNAs [6].

Reactivity of natural R strains varies considerably [13], but the nature of this variability is unknown. Heterochromatic *I*-element-related copies were proposed to play a role in suppression of the hybrid dysgenic syndrome in low reactive R strains [13]. There is also strong evidence that piRNAs from pericentric clusters play a role in the protection against dysgenesis syndrome in *D*. *virilis* [14,15]. The effect of R female age on their reactivity was shown to be mediated by the accumulation and maternal transmission of secondary piRNAs derived from the *I*-element-related copies in ovaries of aged parents [16]. Thus, defective *I*-element copies and piRNAs produced by them play a critical role in the protection against active *I*-element invasion. However, the molecular basis and role of the piRNA system in the natural variation of reactivity of R strains has never been explored. To uncover the natural mechanisms of different efficiencies of TE suppression, we performed a systematic study of a set of *D*. *melanogaster* R strains collected in France in the middle of 20^th^ century, and revealed the crucial role of the piRNA system in variable response to TE invasion. We show that the reactivity of natural R strains negatively correlates with the ancestral *I*-element-specific piRNA content. Exploring the reasons for high level of *I*-specific piRNAs in one of the most *I*-element-resistant R strain, we discovered that natural variation in the efficiency of primary piRNA production in the germline and somatic follicular cells is the most likely reason for enhanced production of ancestral *I*-element piRNAs. Our data suggest that the efficiency of the primary piRNA production varies among natural populations, which can dramatically influence the content of different TEs. piRNA proteins have been shown by several groups to be evolving rapidly under adaptive evolution [17,18,19,20,21]. Our data are the first to demonstrate an important phenotype that might be caused by this kind of variation.

## Results

### Abundance of piRNAs complementary to *I*-elements is correlated with the reactivity of R strains

**Fig 1:**
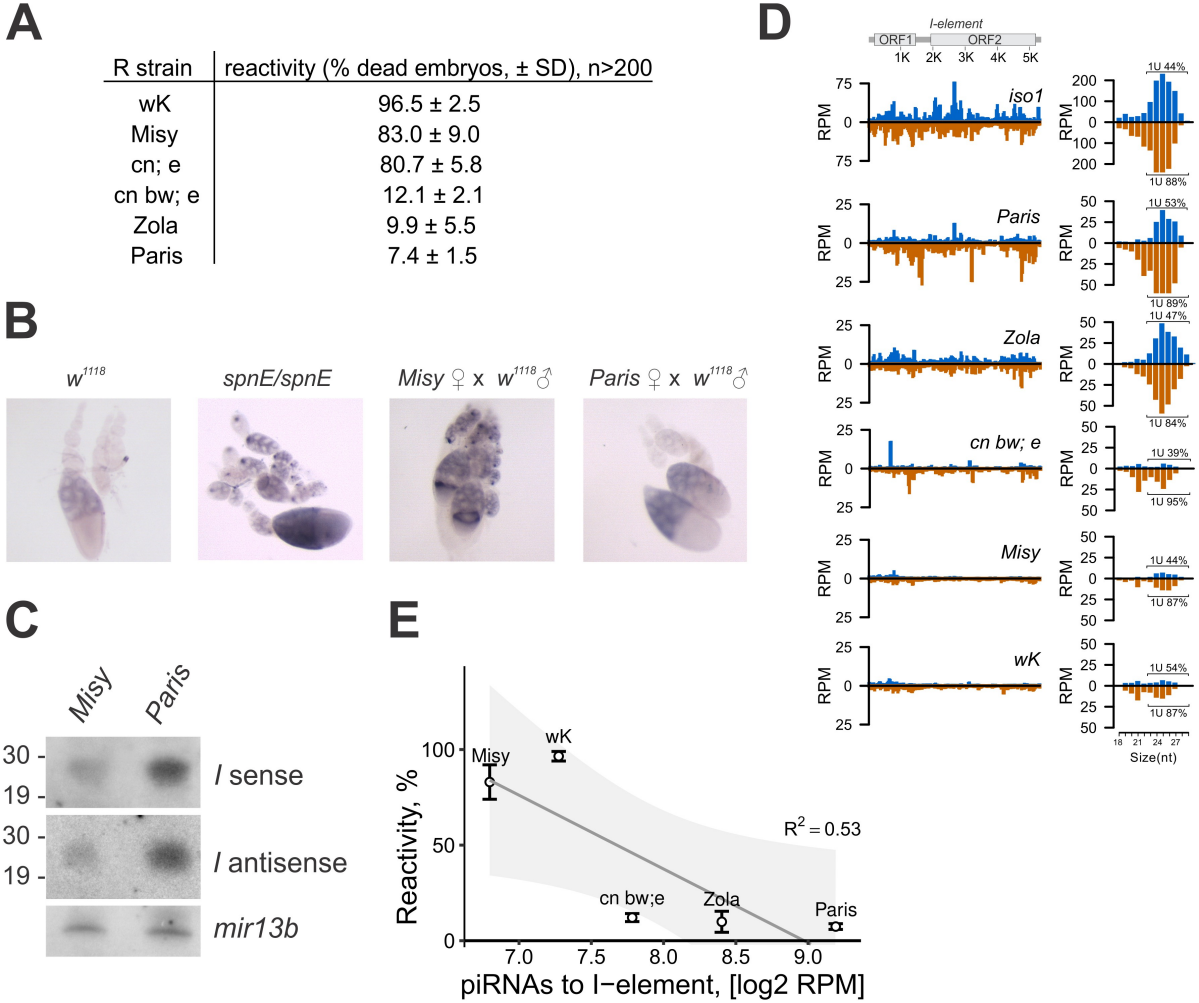
Abundance of *I*-element-specific piRNA correlates with the reactivity of R strains. (A) Reactivity of R strains measured as a percent of non-hatched embryos. Standard deviation (SD) for three replicates is shown; n designates the number of embryos analyzed. (B) Different manifestation of hybrid dysgenesis for *Misy* (strong) and *Paris* (weak) R strain. spnE/spnE is a transheterozygous *spindle-E* mutants (*spn-E*^*1*^*/spn-E*^*hls3987*^*)*. RNA *in situ* hybridization of ovaries with *I*-antisense riboprobe is shown. (C) Weak R strain produces more abundant *I*-element specific piRNA. Northern analysis of the RNA isolated from ovaries of *Paris* and *Misy* strains. Hybridization was done with *I*-element riboprobes to detect sense (I sense) and antisense (I antisense) piRNAs. Lower panel represents hybridization to *mir-13b1* microRNA. P^32^-labeled RNA oligonucleotides were used as size markers. (D) *I*-element specific small RNA mapping to canonical *I*-element. Analysis of ovarian small RNA libraries from strong (*Misy* and *w*^*K*^) and weak (*Paris*, *Zola*, *cn bw;e*) R strains (0–3 mismatches allowed). Reads mapped to the sense strand are shown in blue, and antisense in brown. Length distribution of small RNAs mapping to *I*-element is plotted on the right. Percentages of reads having 1U bias are indicated for each strand (only 24–29 nt reads were considered) (E) Negative correlation between reactivity and *I*-element specific piRNA counts for R strains. Spearman correlation tests: r = -0.90, P-value <0.1. The line depicts the results of linear regression analysis for the level of reactivity and amount of piRNAs. R^2^—adjusted squared R (P-value < 0.1); the grey zone illustrates the 90% confidence interval.

R strains used in the study are of wild origin, collected in France before the 1950`s and were maintained in the collection of Institut de Genetique Humaine (CNRS), Montpellier. The reactivity of chosen R strains varies significantly, as shown in Fig 1A. *I*-element expression in the ovaries of dysgenic females is greater in a cross with the strong R strain, *Misy*, compared to the weak R strain, *Paris*, and is comparable with *I*-element activation in piRNA pathway gene *spn-E* mutant (Fig 1B). In order to understand why R strains differ so much in their ability to protect against transposon invasion, we first estimated the amount of piRNAs specific to *I*-element in the ovaries of R strains. By Northern analysis, we show that *I*-specific small RNAs are significantly more abundant in ovaries of the weak R strain *Paris* than in strong R strain *Misy*; their level in *Paris* is comparable with *I*-specific piRNA content in I strains (Fig 1C, S1 Fig). The differences in the *I*-element-specific small RNA content were verified by sequencing of small RNAs extracted from the ovaries of four R strains. We found that weak-intermediate R strains *Paris*, *Zola*, and *cn bw; e* contain much more *I*-specific small RNAs than *Misy* and an earlier characterized strong R strain, *w*^*K*^ (Fig 1D). Most of the *I*-specific small RNAs are 24–29 nt long and show the characteristic nucleotide bias of piRNA species (1U). Reactivity negatively correlates with the *I*-element-specific piRNA content (Spearman test, r = -0.9, P-value <0.1; Fig 1E).

To verify that the difference in *I*-element piRNA abundance between strong and weak R-strains is statistically significant, we have performed the differential expression analysis of piRNAs. We processed the pairs of small RNA-seq from strong (*Misy* and *w*^*K*^) and weak (*Paris* and *Zola*) R strains as pseudo-replicates. Indeed, we found that *I*-element piRNAs are significantly more abundant in weak R strains than in strong ones (log_2_ fold change = 2.33, P-value = 9.8e-4) (S1 Table).

**Fig 2:**
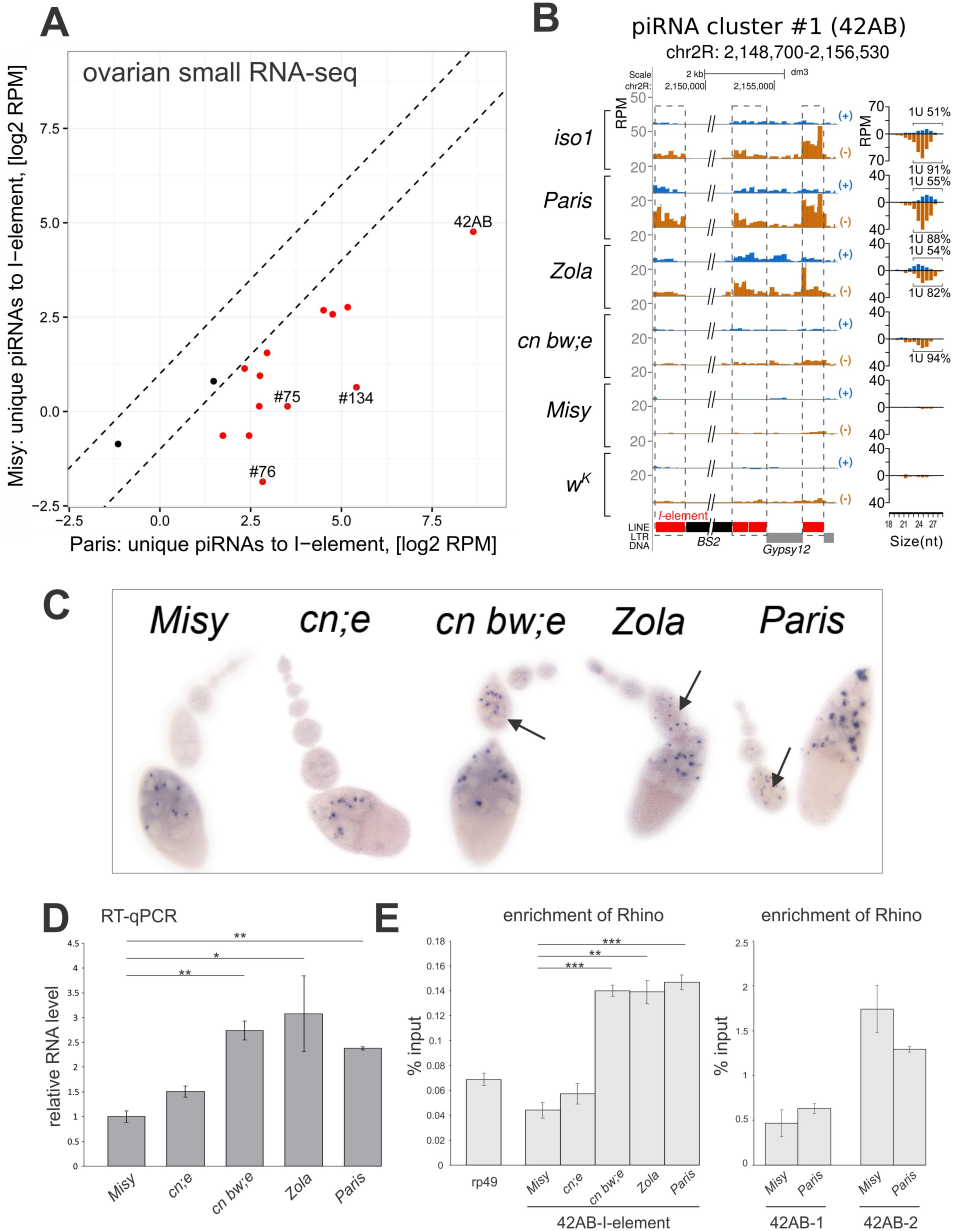
Transcription, chromatin structure, and piRNA production of *I*-element fragments located within piRNA clusters in R strains. (A) Scatter plot shows normalized single-mapped small RNAs mapping to *I*-element remnants from *I*-element containing piRNA clusters in *Paris* versus *Misy* ovaries. (B) Single-mapped small RNAs specific to the *I*-element fragments within cluster #1 (*42AB*) in R strains and I strain *iso-1*. Small RNA mapping was done to the reference genome. Sequencing of this region in R strains (S2B Fig) revealed minor variations that were not considered in alignments. Red and grey boxes correspond to the *I*-element or other TE fragments, respectively; dashed lines indicate matching of the small RNA plots to the *I*-element fragments. Reads mapped to the sense strand are shown in blue, and antisense in brown. (C) *In situ* RNA hybridization with transcripts of the *42AB* piRNA cluster (*I*-element region) in ovaries of R strains. Arrows show accumulation of piRNA precursors at earlier stages of oogenesis in weak R strains. (D) RT-qPCR analysis of piRNA precursor expression within the regions of *42AB* piRNA cluster containing *I*-element remnants (*42AB-I*) in ovaries of R strain females obtained from young parents. (E) Rhi ChIP-qPCR on ovaries of R strain females obtained from young parents at the regions of the *42AB* piRNA cluster containing *I*-element remnants (*42AB-I*) or harboring other TEs (*42AB-1*, *42AB-2*). Asterisks indicate statistically significant differences relative to *Misy* (* P < 0.05 to 0.01, ** P < 0.01 to 0.001, *** P < 0.001, t-test).

piRNA clusters produce the majority of TE-specific piRNAs in *Drosophila* ovaries. Distinct piRNA pathways function in the germline and ovarian somatic cells. In follicular cells, primary piRNAs are generated from the single strand precursors mainly transcribed from uni-strand *flamenco* piRNA cluster to silence retroviral elements such as *gypsy*, *ZAM* and *Idefix* [1,22,23]. Dual-strand piRNA clusters that generate piRNAs corresponding to both genomic strands suppress a broad spectrum of TEs in the germline [1,24]. In the germline, primary piRNAs produced by the piRNA clusters are amplified through the ping-pong amplification loop [1,25]. Ancestral *I*-element fragments reside in the dual-strand piRNA clusters [6]. Therefore, we tested whether differences in the level of cluster-derived *I*-specific piRNAs in R strains correlated with different reactivity. To address this, we performed a genome-wide comparison of the content of single-mapped small RNAs corresponding to *I*-element fragments that map to different piRNA clusters [1], between a strong R strain, *Misy*, and a weak R strain, *Paris*. We found that the amount of such small RNAs is much higher in the *Paris* strain than in *Misy* (Fig 2A). At first, we looked specifically at the *I*-element fragments within *42AB*, which is the strongest piRNA cluster in *D*. *melanogaster* [6]. A normalized amount of such single-mapped piRNAs was higher in weak R strains (Fig 2B). Reactivity negatively correlates with the content of single-mapped *I*-element-specific piRNAs from *42AB* (Spearman test, r = -0.9, P-value <0.1; S2A Fig). Similarly to cluster *42AB*, we found that the content of single-mapped small RNAs corresponding to the *I*-element fragments within piRNA clusters 75, 76, and 134 was higher in the weak R strain than in the strong R strain (S3A Fig). PCR of genomic DNA followed by sequencing confirmed that in all strains the *I*-element fragments were intact within piRNA clusters (S2B and S3B Figs). *BS2* and *Rt1b* insertions within *I*-element fragments were identified in *42AB* and #134 piRNA clusters, respectively, in some R strains (S2B and S3B Figs). However, these insertions do not cause global changes in the number of small RNAs mapping to these loci (Fig 2B, S3A Fig).

In summary, we found the negative correlation between reactivity of R strains and the amount of ancestral *I*-element specific piRNAs. Therefore, we set out to explore the nature of these differences in piRNA production.

### Transcription, chromatin structure, and piRNA production by piRNA clusters in R strains

To compare the expression of piRNA precursors generated by piRNA clusters in different R strains, we chose a region of the *42AB* cluster that contains a fragment of an *I*-element. We designed a 42AB-I probe corresponding to this region and performed *in situ* RNA hybridization on the ovaries of R strains. In weak R strains, signals were stronger and were detected in the nuclei of nurse cells at the early stages of oogenesis, in contrast to strong R strains, where staining was observed only at later stages (Fig 2C). The probe has 78% identity (442 bp, e-value = 5e-109) with a canonical *I*-element and could detect not only *42AB* transcripts, but also some other ancestral *I*-element transcripts. Thus, this result suggests that expression of *I*-element remnants including those localized in the *42AB* cluster is stronger in weak R strains.

To confirm this observation, we studied the expression of this region by RT-qPCR of total ovarian RNA from R strains. Primers used for RT-PCR and ChIP-qPCR analyses of 42AB-I-element were designed using sequences of all studied R strains (S2B Fig). To avoid aging effects that considerably affect reactivity and expression of *I*-related heterochromatic copies [16,26], 3-day-old females obtained from young parents that were also obtained from young parents (and so on, for at least seven generations) were used for analysis. The steady-state level of the piRNA precursor transcripts from this region was significantly higher in weak R strains (Fig 2D). A similar result was obtained when 3-day-old females from parents of mixed ages were used (S4A Fig). These data show that the expression level of the *I*-element-specific piRNA precursors is an intrinsic characteristic of different R strains with different reactivity. We did not detect a difference in the expression level of two other regions of the *42AB* cluster, harboring fragments of active TEs (S4B Fig).

It is important to note that *Gypsy*12 and *Cr*1 TE fragments next to the *I*-element within *42AB* and #134 piRNA clusters, respectively (Fig 2B, S3A Fig), also produce very few single-mapped piRNAs in the *Misy* strain, which allowed us to suggest that these TE fragments are likely part of the same precursor transcripts as the *I*-element remnants. One may suggest that such transcripts would be targeted by low abundant *I*-element piRNAs, resulting in a low level of 3’-directed Zucchini-dependent piRNA production [27,28].

The germline-specific HP1 homolog, Rhino (Rhi), is essential for piRNA production by dual-strand piRNA clusters, suggesting its putative role in piRNA precursor transcription [29,30,31]. We performed Rhi ChIP-qPCR and observed that the region of the *42AB* cluster containing the *I*-element fragments shows significantly higher Rhi occupancy in the weak R strains (Fig 2E). Equally high enrichments of Rhi were observed at *42AB* regions devoid of *I*-element fragments in the strong R strain *Misy* and weak R strain *Paris* (Fig 2E). Thus, *I*-element remnants located within piRNA clusters produce more piRNAs in the weak R strains than in the strong R strains, which is in accordance with a high level of piRNA precursor transcription as well as Rhi binding to these regions.

It is believed that piRNA cluster transcripts are processed into primary piRNAs, which further increase the abundance and diversity of piRNAs by engaging in an amplification process with the transposon and piRNA cluster transcripts, or Zucchini endonuclease dependent, phased piRNA production [1,16,25,27,28]. Biogenesis of piRNAs represents a classic feedback regulatory mechanism in which changes in piRNA production at any stage can be amplified or suppressed at subsequent steps. We tested several models that could explain the difference in the content of *I*-element piRNAs in R strains. If a higher level of *I*-element piRNAs in weak R strains are *I*-element-specific, it would be easily explained by the presence of extra *I*-element fragments within piRNA clusters in these strains. Alternatively, general inter-strain differences in the efficiency of piRNA processing or maternal transmission of piRNAs could potentially affect *I*-element and other TE piRNA abundance. We suggest that in natural R strains different causes, or combinations thereof, may lead to low reactivity. For further in-depth analysis of the natural variations in the efficiency of anti-transposon response, we chose two R strains from the opposite sides of the reactivity spectrum: a strong R strain, *Misy*, and the *Paris* strain, which demonstrates the lowest reactivity to the *I*-element (Fig 1A).

### Genomic content of *I*-element fragments and maternal deposition of piRNAs is similar in two R strains with different production of *I*-element-specific piRNAs

**Fig 3:**
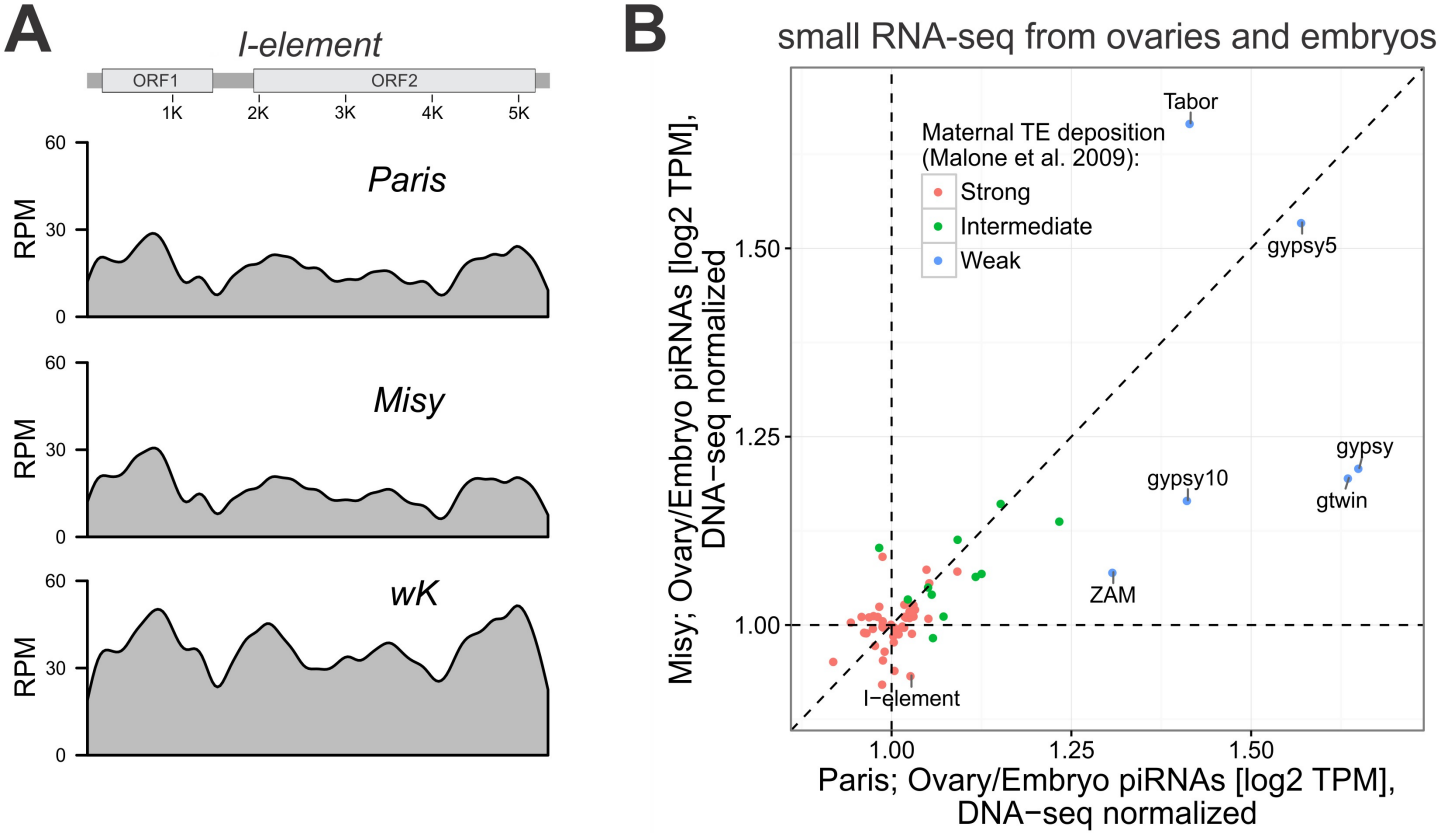
Genomic content of *I*-element fragments and maternal deposition of piRNAs is similar in R strains with different reactivity. (A) Analysis of genomic DNA libraries. *I*-element genomic sequence coverage revealed no difference in the *I*-element copy number between *Paris* and *Misy* strains. (B) Scatter plot shows ratio of normalized TE-specific piRNAs (24–29 nt reads were considered) in ovaries to that of 0-2-hour-old embryos in *Paris* and *Misy* strains. The piRNA expression was additionally normalized to the log_2_-transformed genomic abundance of the corresponding TEs (RPM, reads per million). The color of dots indicates the type of TEs according to its capacity to maternal deposition in embryos according to [24]. TPM, transcripts per kilobase per million.

It was shown previously that piRNA abundance correlates with TE copy number both genome-wide and within piRNA clusters [32]. It is possible that differences in the abundance of *I*-element-specific piRNAs in R strains can be explained by the fact that the genomes of weak R strains accumulate more *I*-element remnants located within endogenous piRNA clusters and produce more *I*-element-specific piRNAs overall. To test this hypothesis, we sequenced the genomes of both *Paris* and *Misy* strains. At first, we looked specifically at genomic sequences corresponding to *I*-elements and found them to be very similar (Fig 3A). The analysis of TE copy abundance using DNA-seq data did not reveal a significant difference in *I*-element occupancy between *Paris* and *Misy* genomes (P-value = 0.74, two-sided Wilcoxon rank sum test). At the same time, genomes of both *Paris* and *Misy* strains have much fewer *I*-elements compared to the *w*^*K*^ strain (P-value < 1.0e-15, two-sided Wilcoxon rank sum test). Moreover, the *I*-element remnant landscape is similar in the genomes of *Paris* and *Misy* strains (S2 Table) which is in accordance with previously reported data relating to the fixation of older TE insertions [33], and may indicate the common origin of these strains. A few strain-specific *I*-element insertions predicted in *Paris* and *Misy* genomes locate at the regions producing a negligible number of unique small RNAs in both strains (S5 Fig). Many of the *I*-element insertions in the genome of the strong R strain *w*^*K*^ were absent from the reference I strain *iso-1* as well as other R strains (S2 Table). Thus, strong differences in reactivity and piRNA production cannot be explained by variation in the number of ancestral *I*-element remnants present in different R strains. Similar to our observation, non-significant relationship between the incidence of hybrid dysgenesis and paternal P-element dosage was reported [34]. Nevertheless, we cannot completely exclude the possibility that some actively transcribed *I*-element fragments hidden in the unassembled heterochromatin could still contribute to the piRNA production in weak R strains.

The maternal pool of piRNAs launches processing of the master locus transcripts into piRNAs in progeny [6]. It is possible that transmission of maternal piRNAs might be more efficient in the R strain *Paris*, that could lead to the enhanced production of piRNAs in general in this strain. To test this, we analyzed the repertoire of piRNAs in 0-2-hour-old embryos, before zygotic activation of transcription (small RNA-seq from embryos), and compared the piRNA ratio in ovaries to that in early embryos for two R strains, *Misy* and *Paris*, normalized to the TE copy number (Fig 3B). We did not observe a higher level of maternal transmission for piRNAs corresponding to an *I*-element or any other TEs expressed in the germline of strain *Paris* compared to *Misy* (Fig 3B, pink and green dots). However, accumulation of piRNAs corresponding to TEs with predominant expression in follicular somatic cells (Fig 3B, blue dots) is observed in the ovaries of the *Paris* strain, indicating that these transposons produce more piRNAs. Comparison of the content of TE-specific piRNAs between *Misy* and *Paris* 0-2-hour-old embryos shows that *I*-element small RNAs are more abundant in embryos of *Paris* strain than in *Misy*, which is in agreement with ovarian small RNA data (S6 Fig).

Thus, the strong suppression of the *I*-element in the *Paris* R strain could not be explained by either the accumulation of *I*-element remnants nor enhanced maternal deposition of piRNAs in the *Paris* strain compared to *Misy*. Instead, it is likely caused by the enhanced production of piRNAs from the same *I*-element remnants scattered along piRNA clusters.

### Variation in the abundance of primary piRNAs in R strains

Two R strains with dramatically different reactivity have the same *I*-element fragment copy number, but these copies generate more piRNAs in the weak R strain *Paris* than in the strong R strain, *Misy*. We hypothesized that in *Paris* production of primary piRNAs is enhanced in general and is not restricted to any particular TE, including the *I*-element. However, with the exception of *I*-element fragments, it is difficult to examine primary processing in the germline: the amount of primary piRNAs corresponding to active transposons is confounded by the presence of secondary piRNAs derived from the ping-pong piRNA amplification loop [1,25]. A characteristic of the ping-pong mechanism is the number of complementary piRNA pairs with a 10-nucleotide overlap between their 5’ ends [1,25]. Comparison of the total piRNA content between *Misy* and *Paris* strains did not detect global differences (S7A Fig). Ping-pong piRNA profiles of TEs expressed in the germline are also highly similar in R strains (S7B Fig). This indicates that a general limitation of the germline primary piRNA biogenesis in the strong R strain, if any, can be compensated by the ping-pong amplification for most of the active TEs. Accordingly, we did not observe variations in the expression of piRNA precursors, or Rhi enrichment at the regions of the *42AB* locus harboring fragments of TEs active in either strain (Fig 2E, S4B Fig).

**Fig 4:**
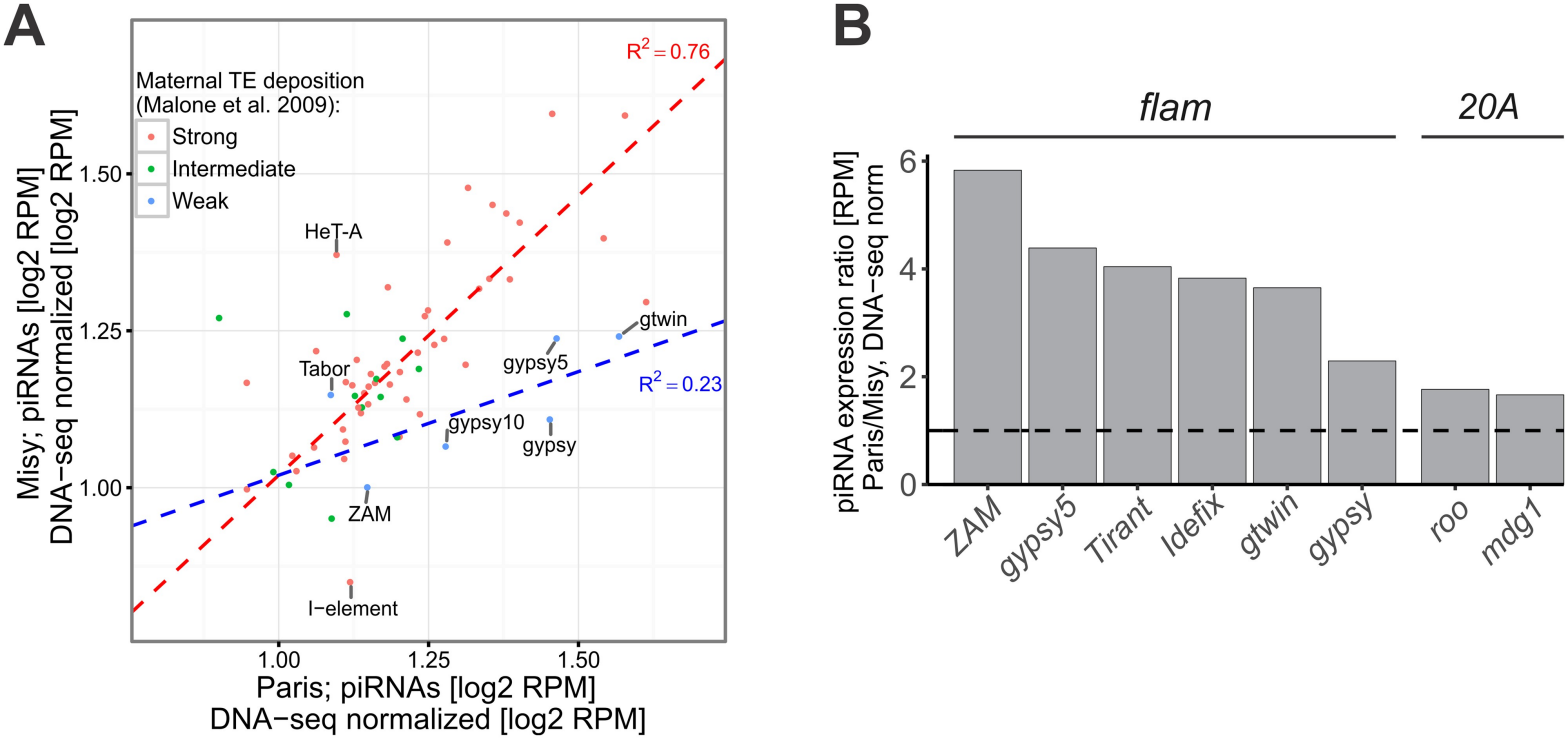
Uni-strand piRNA clusters produce more piRNAs in the *Paris* than in the *Misy* strain. (A) Scatter plot of log_2_-transformed and RPM-normalized small RNA expression in ovaries of *Paris* and *Misy* strains. piRNA expression was additionally normalized to the log_2_-transformed and RPM-normalized genomic abundance of the corresponding TEs estimated as number of DNA-seq reads mapped to the canonical TE sequences. The color of dots indicates the type of TEs according to its capacity for maternal deposition in embryos according to [24]. Dashed lines depict the results of linear regression analysis for TEs with high maternal deposition (genuine germinal TEs, red) and for TEs with weak maternal deposition (genuine somatic TEs, blue). R^2^—adjusted squared R (P-value < 0.1). (B) Relative abundance (*Paris/Misy*) of a normalized number of single-mapped small RNA reads (RPM, 24–29 nt reads were considered) mapping to TE copies located within uni-strand piRNA clusters #8 *(flamenco*, expressed in follicular cells) and #2 (*20A*, expressed in the germline).

Next, we decided to look at the variation in the content of piRNAs derived from uni-strand piRNA clusters, where piRNAs originate from one genomic strand and are not subjected to secondary amplification. Comparison of the abundance of TE-specific piRNAs normalized to copy number between *Misy* and *Paris* shows that TEs expressed predominantly in follicular cells [24] produce more piRNAs in *Paris* (Fig 4A). A ping-pong-independent piRNA pathway operates in *Drosophila* follicular cells [24]. Therefore, we decided to determine differences in primary piRNA processing between *Drosophila* strains by analyzing follicular piRNAs.

To compare cluster-derived piRNAs, we focused on the piRNAs single-mapped to the TEs from the major uni-strand somatic piRNA cluster *flamenco* (cluster #8). We detected more piRNAs corresponding to such TEs (normalized to copy number) in *Paris* compared to *Misy* (Fig 4B). piRNA cluster #2 located at *20A* region of X chromosome is the only known uni-strand cluster that is expressed in the germline and produces primary piRNAs [30]. We compared piRNA production by cluster *20A* in *Misy* and *Paris*. Since we failed to assemble the genomic sequence of the entire *20A* cluster using DNA-seq data, we analyzed the regions enriched by single-mapped piRNAs, comprised of highly damaged fragments of *mdg1* and *roo* TEs. Comparison of single-mapped piRNAs generated by distinct regions of *20A* (normalized to copy number) revealed their increased abundance in ovaries of *Paris* compared to *Misy* (Fig 4B). We also observed decreased content of single-mapped piRNAs (normalized to the TE copy number) generated by TE fragments located in *flamenco* and *20A* uni-strand piRNA clusters in ovaries of the strong R strain *w*^*K*^, compared to the *Paris* strain (S8 Fig).

**Fig 5:**
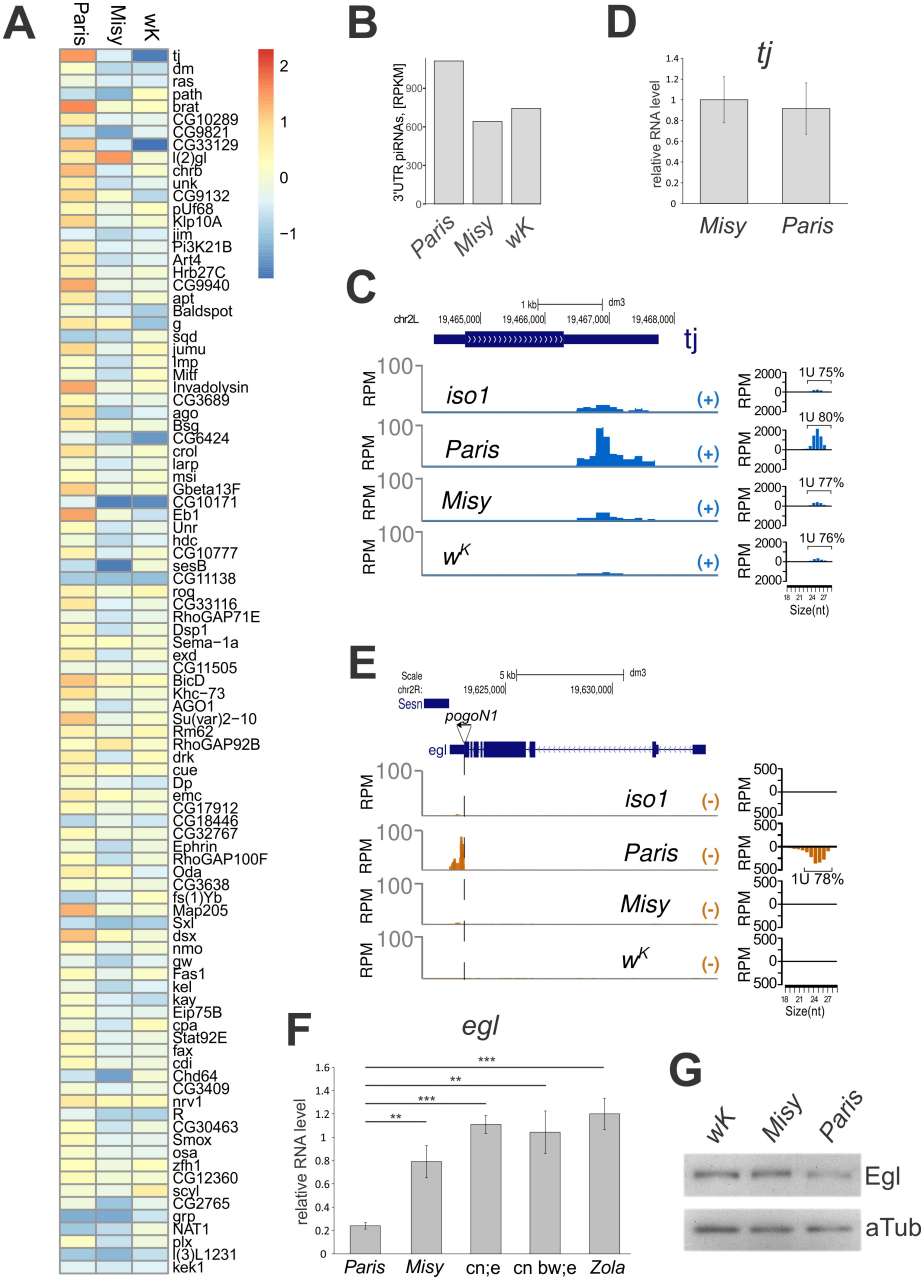
Polymorphism in the abundance of the 3’–genic piRNAs in R strains. (A) Heatmap shows RPKM (Reads Per Kilobase Million, only 24–29 nt reads were considered) corresponding to the most active 3’ genic piRNA clusters in follicular cells [35] in *Paris*, *Misy* and *w*^*K*^ strains relative to *iso-1* strain. Heatmap is sorted for decreasing RPKM count for 3' genic piRNA clusters in *Paris* strain. A comparison of genome sequences revealed no significant differences in the regions comprising six major genic piRNA clusters in these R strains (S9 Fig). (B) Abundance of small RNA reads (RPKM, 24–29 nt reads were considered) mapping to the genic piRNA clusters [35] in *Paris*, *Misy* and *w*^*K*^. (C) Profile of small RNA density at *tj* locus in ovaries of *Misy*, *Paris*, *w*^*K*^ and *iso-1* strains. Length distribution of *tj* small RNAs is plotted on the right. Percentages of reads having 1U bias are indicated. (D) RT-qPCR analysis of *tj* expression in ovaries of R strains. (E) Profile of small RNA density at the *egl* locus in ovaries of *iso-1*, *Misy*, *w*^*K*^ and *Paris* strains. Length distribution of *egl* small RNAs is plotted on the right. Percentages of reads having 1U bias are indicated. (F) RT-qPCR analysis of *egl* expression in ovaries of R strains. Asterisks indicate statistically significant differences relative to *Paris* (* P < 0.05 to 0.01, ** P < 0.01 to 0.001, *** P < 0.001, t-test). (G) Western blot of *w*^*K*^, *Misy* and *Paris* ovary extracts probed with antibodies against Egl and α-tubulin. The level of Egl protein is 1.5±0.2 fold higher in ovaries of strain *Misy* than in *Paris*. The value is mean±SD for two anti-Egl western blot experiments with three dilutions of ovary extracts normalized to α-tubulin.

Next, we compared piRNA production by genic 3’UTR (untranslated region) piRNA clusters [35] between *Paris*, *Misy* and *w*^*K*^ and observed that the majority of the 3’ genic piRNA clusters including *traffic jam (tj*), which is the strongest genic piRNA cluster in follicular cells [36], produce more piRNAs in *Paris* (Fig 5A and 5B). We confirmed this by Northern blotting of *tj* small RNAs (S9A Fig). A comparison of genome sequences revealed no significant differences in the regions encompassing major genic piRNA clusters in *Paris*, *Misy* and *w*^*K*^ strains (S9B and S9C Fig). Cluster analysis of piRNA production by 3’UTR piRNA clusters in R strains and in 16 RAL strains from the Drosophila melanogaster Genetic Reference Panel (DGRP) [37,38] shows strong inter-strain differences in the efficiency of genic piRNA processing. *Paris* strain belongs to the group producing most abundant 3’UTR piRNAs (S10 Fig).

We discovered a natural variability in genic piRNA abundance and decided to explore the influence of this variability on the level of mRNAs that serve as the piRNA precursors. Stronger piRNA processing of *tj* mRNA in *Paris* does not significantly affect the steady state level of *tj* mRNA (Fig 5C and 5D). In addition, we discovered a potent 3’ genic piRNA cluster in the *egalitarian (egl)* 3’UTR that originated as a result of a *pogoN1* transposon insertion in *Paris*, leading to the production of abundant piRNAs collinear to the *egl* mRNA (Fig 5E, S9A Fig). Egl is an RNA-binding protein essential for the localization of transcripts during oogenesis and early development [39]. Expression analysis of the *egl* gene in different strains has shown a decrease in the steady-state level of *egl* mRNA as well as Egl protein in ovaries of strain *Paris* (Fig 5F and 5G), which is likely a result of mRNA degradation by piRNA processing. Thus, we demonstrate that piRNA production *per se* in some cases can affect mRNA stability and cause moderate effects on the expression of genes harboring 3’UTR piRNA clusters.

We conclude that production of the primary piRNAs in the germline and follicular cells is more efficient in *Paris*. We suggest that low reactivity of the *Paris* strain likely results from high efficiency of primary piRNA production in general rather than only *I*-element-specific piRNAs. What could be the reason for natural variability in primary piRNA production?

### Analysis of amino acid polymorphism in the piRNA pathway components

piRNA factors are rapidly evolving proteins characterized by an increased rate of amino-acid evolution [17,18,19,20,21]. Allelic polymorphism of piRNA biogenesis factors could influence piRNA biogenesis. Taking into account the greater abundance of primary piRNAs both in the germline (*I*-element and *20A* uni-strand piRNA cluster) and in follicular cells (*flamenco* and 3’UTR genic clusters) in *Paris*, one may suggest that these effects are mediated by a mutation of a piRNA factor/factors common for both ovarian tissues. High intra-specific variability in the level of the piRNA pathway gene transcripts was revealed in *D*. *simulans* transcriptome studies [17,40]. Using western-blotting, we did not detect differences in the protein expression of the main small RNA pathway factors in ovarian extracts from *Misy* and *Paris* strains (S11 Fig). However, this leaves the possibility that studied *Drosophila* strains contain allelic variants of some piRNA biogenesis factors characterized by different biochemical activity that affect primary processing. We compared the genomes of *Misy*, *Paris* and 16 fruit fly RAL strains from the DGRP project [37] to look for possible allelic polymorphisms in genes encoding piRNA biogenesis factors. As expected, all strains display numerous polymorphic sites in the piRNA factors, including functionally significant amino acid substitutions; some of them are specific for *Paris* (S3 Table). We believe that some allelic variant or a combination of several variants is responsible for an unusually high efficiency of primary piRNA production in this strain. Our findings provide a strong basis for further in-depth analysis of the naturally occurring polymorphism of the piRNA system and its role in adaptive genome defense.

### A weak R strain *Paris* contains a dramatically decreased telomeric element *HeT-A* copy number

During the analysis of TE-specific small RNA in R strains, we noticed an extremely low read number of telomeric retrotransposon *HeT-A* piRNAs in *Paris* (Fig 4A). The telomeres of *D*. *melanogaster* consist of the specialized telomeric retrotransposons *HeT-A*, *TART* and *TAHRE* [41,42,43,44]. *HeT-A* is a main structural component of *Drosophila* telomeres represented by about 30 complete elements per diploid genome [42,45] while *TART* and *TAHRE* are represented by several copies, but are not found in every telomere [41].

**Fig 6:**
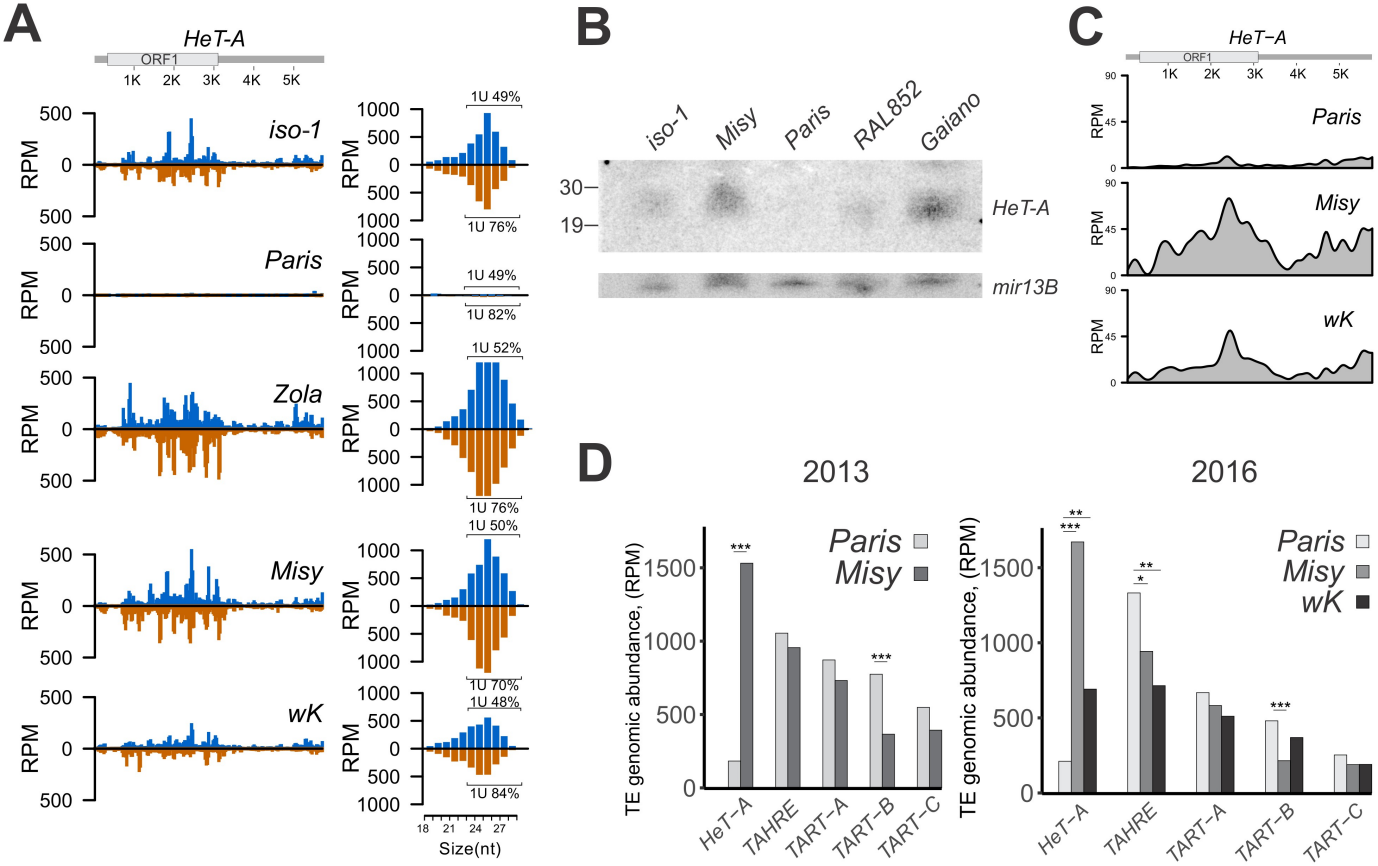
Characteristics of telomeres in the *Paris* strain. (A) The number of *HeT-A*-specific small RNA is dramatically reduced in strain *Paris*. The density of small RNAs along the *HeT-A* canonical sequence is shown. Length distribution of *HeT-A* small RNAs is plotted on the right. Percentages of reads having 1U bias are indicated for each strand (only 24–29 nt reads were considered). Most of the *HeT-A*-specific small RNAs are 24–29 nt in size and show the characteristic 1U bias of piRNA species. (B) Northern analysis of the RNA isolated from the ovaries of *iso-1*, *Misy*, *Paris*, RAL-852 and *GIII* strains. Hybridization was done with the *HeT-A* antisense riboprobe. Lower panel represents hybridization to the *mir-13b1* microRNA. (C) Analysis of genomic DNA libraries. Telomeric retrotransposon *HeT-A* genomic sequence coverage in *Paris*, *Misy* and *w*^*K*^ strains. (D) The number of *HeT-A* genomic reads is dramatically reduced in *Paris*. Normalized number of genomic reads (RPM) mapping to the canonical telomeric retrotransposons in R strains. Analysis of mate-paired (made in 2013) and paired-end (made in 2016) *Paris* and *Misy* genomic DNA libraries. Paired-end genomic sequences of *w*^*K*^ were described previously [5]. P-values: * <1e-5, ** <1e-10, *** <1e-15, two-sided Wilcoxon rank sum test).

Ovarian small RNA mapping to the canonical *HeT-A* sequence (Fig 6A) and Northern analysis of small RNAs (Fig 6B) confirmed that the number of *HeT-A*-specific small RNAs is dramatically reduced in *Paris*. Genomic sequence coverage shows that the *HeT-A* copy number in *Paris* is substantially lower than in *Misy* and *w*^*K*^ (Fig 6C and 6D). The analysis of TE copy abundance using DNA-seq data revealed a significant difference of *HeT-A* occupancy between *Paris* and *Misy or w*^*K*^ genomes (Fig 6D). To evaluate relative *HeT-A* copy number, we performed PCR on genomic DNA of different strains (S12 Fig) and confirmed that in the genomes of two independent *Paris* substrains, the *HeT-A* copy number is dramatically lower than in *Misy* and other studied strains, while the *HeT-A* copy number did not correlate with reactivity (S12 Fig). A relationship between the abundance of *HeT-A* piRNA, as detected by Northern blot (Fig 6B) and *HeT-A* copy number (S12 Fig), was observed. In the strain *Gaiano*, which is characterized by increased *HeT-A* copy number [46,47], abundant *HeT*-A-specific piRNAs were detected by Northern blotting (Fig 6B).

The strain *Paris* possesses a lower *HeT-A* copy number compared to the *D*. *melanogaster* strain RAL-852, that was previously shown to have the lowest *HeT-A* copy number in the DGRP collection [48]. We also failed to detect *HeT-A* in *Paris* using DNA FISH on polytene chromosomes of salivary glands (S13 Fig). A comparison of genome sequence data obtained in 2013 and 2016 (Fig 6D) revealed a stable heritable low *HeT-A* copy number in *Paris*. The analysis of TE copy number and genomic sequence coverage on other telomeric retrotransposons, *TART* and *TAHRE*, did not show a dramatic difference between the *Paris* and *Misy* strains, although slightly increased copy number of these elements may indicate that they can partially compensate for *HeT-A* loss in *Paris* (Fig 6D, S14A Fig). Abundant *TART*-specific small RNAs are revealed in ovaries of *Misy* and *Paris* (S14B Fig).

Evolutionary conserved cooperation of autonomous and non-autonomous telomere-specific retrotransposons may be explained by distribution of different roles among elements [49]. Most likely, *TART* provides reverse transcriptase activity for non-autonomous *HeT-A* that serves as a main structural component of *Drosophila* telomeres [50]. Indeed, *HeT-A* and *TART* are characterized by different copy number, structure and patterns of transcription [42,45,50,51]. The strain *Paris* is an excellent model to study the roles of different telomeric elements and particularly *HeT-A* in genome stability. We speculate that elimination of *HeT-A* in *Paris* may be the direct consequence of enhanced primary piRNA production in this strain (see Discussion).

## Discussion

In this study, we found that natural variations in the level of TE-specific piRNAs can lead to dramatic differences in the protection against *I*-element transpositions in I-R dysgenic crosses. It is the first report of age-independent strain-specific variation in the general efficiency of piRNA production; the previously published examples described distinct piRNA cluster-specific variations [3,52].

A correlation was discovered between the hybrid dysgenic abnormality rate and the level of piRNAs corresponding to the ancestral *I*-element fragments in natural *D*. *melanogaster* R strains lacking an active *I*-element. Additionally, a negative relationship between reactivity and transcription of the piRNA precursors as well as Rhi binding to the piRNA cluster regions containing *I*-element fragments was observed. An estimation of the level of piRNA cluster transcripts upon Rhi depletion led to conflicting results [29,31], leaving a question about the exact role of Rhi in the piRNA cluster activity. From our data we can conclude that primary piRNA production, Rhi enrichment, and transcription of the piRNA-producing locus are closely related to each other in the germline-specific dual-strand piRNA clusters. This correlation was observed only for the ancestral *I*-element fragments in R strains lacking active *I*-elements. The effect was noticeable because the primary *I*-element piRNAs were not masked by the abundant secondary piRNAs.

The transposition rate of TEs is controlled by distinct mechanisms, including regulation of promoter activity, chromatin structure, splicing, and small RNA pathways [2,51,53,54,55,56]. In some cases, the factors capable of inducing loss of transposition control are unclear. For example, a high level of *hobo* and *I*-element transpositions in the genome of the reference *D*. *melanogaster* strain *iso-1* is observed despite normal production of *hobo*- and *I*-element-specific piRNAs in the ovaries of this strain [5,57,58]. Molecular mechanisms underlying the accumulation of actively transposed *copia* retrotransposon in the genomes of inbred *D*. *melanogaster* strains also remain unknown [59,60]. In dysgenic crosses, maternally transmitted pools of piRNAs play a critical role in the suppression of paternally inherited TEs [6]. However, the nature of the different responses to transposon invasion in different natural strains is poorly understood. Here, we have shown that variation in the ancestral *I*-element-specific piRNA content is responsible for the different manifestations of I-R hybrid dysgenesis mediated by *I*-element mobilization in natural populations. I-R hybrid syndrome represents a simple model of intraspecific hybrid dysgenesis, in which a maternal pool of piRNAs targets the paternally inherited TEs. However, *I*-element mobilization *per se* does not explain the occurrence of the sterility of dysgenic females. The important role of the systemic perturbation in the production of piRNAs including genic piRNAs in the dysgenic gonads has been discussed [14,15]. Our data demonstrating the variation in the abundance of distinct types of piRNAs including ovarian somatic species in natural strains, involved in hybrid dysgenic crosses, agree well with this idea.

The cause of a higher production of *I*-element-specific piRNAs in weak R strains remains unclear; most likely, different mechanisms affect the level of *I*-specific piRNAs in different R strains, which requires deep genome-wide analysis of each strain. Using two R strains, a strong and a weak one, we investigated the putative factors responsible for this phenomenon. We were only able to link strong suppression of *I*-element to enhanced production of primary piRNAs in the weak R strain, *Paris*. We did not detect extra *I*-element fragments capable of producing abundant piRNAs in the genome of *Paris*, however, we do not exclude the possibility that some actively transcribed *I*-element fragments hidden in the heterochromatin could contribute to the piRNA production in *Paris* or in other weak R strains.

The hypothesis about stronger transmission of maternal piRNAs in the *Paris* strain was also rejected since we did not reveal any differences between weak and strong R strains. Instead, we detected inter-strain differences in the levels of primary piRNA production and piRNA precursor expression under controlled aging and temperature conditions, suggesting that natural variation in piRNA production efficiency is responsible for the observed differences.

We speculate that production of primary piRNAs may be more efficient in *Paris* in general due to systemic changes in the piRNA pathway that provide a high level of the ancestral *I*-element piRNA production as well as enhanced production of other primary piRNAs in germ and somatic ovarian cells. We did not reveal quantitative differences in the expression of several piRNA factors or any evidence for alteration in their activity. Certain allelic variants or combinations thereof may be responsible for the higher level of primary piRNAs found in *Paris*. Variation in TE diversity and their copy number requires adaptive evolution of the piRNA machinery. Indeed, strong evidences of rapid positive selection within a core set of piRNA genes within *Drosophila* species were reported by different groups indicating an arms race between the piRNA pathway and TEs [18,19,20,21].

**Fig 7:**
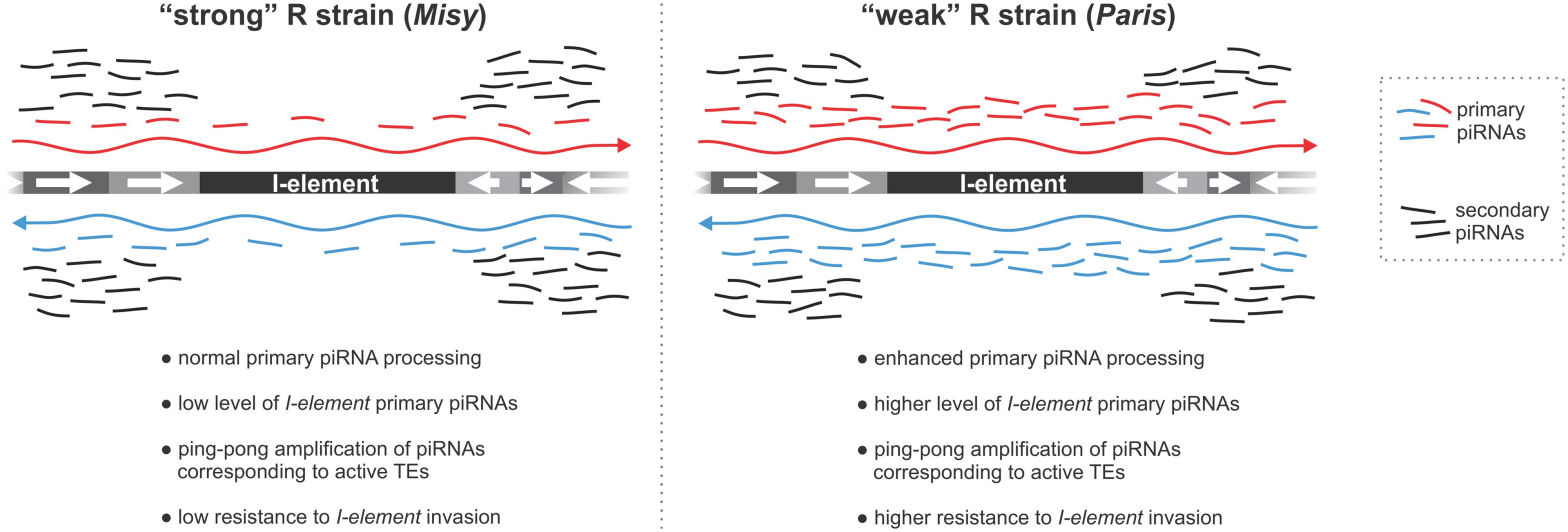
A model explaining the mechanism of higher resistance against the *I*-element in the R strain *Paris* compared to another R strain, *Misy*, in dysgenic crosses. In *Paris*, stronger primary processing of piRNA cluster transcripts in ovarian somatic cells and in female germline generates a bigger pool of primary piRNAs, including *I*-element specific piRNAs, compared to *Misy*. For most active TEs this difference is masked by abundant secondary piRNAs, which arise as a result of the ping-pong amplification loop. For the *I*-element, however, additional primary piRNAs in *Paris* are capable of inducing more efficient suppression of *I*-element in dysgenic crosses. The occurrence of ping-pong between piRNA cluster transcripts and the existence of secondary piRNAs, specific to non-active transposons including the *I*-element [16], is omitted from the scheme for the sake of simplicity.

piRNA pathway genes also exhibited large variation in transcript levels in wild-type strains of *D*. *simulans* [17]. Moreover, TE transcript level was shown to be negatively correlated with piRNA pathway gene expression [40]. However, no relationship between TE abundance and the increasing rate of amino-acid evolution of the piRNA pathway factors was revealed in the *Drosophila* genus [61]. Instead, improved codon usage increasing the translational efficiency of the piRNA machinery was correlated with TE abundance in *Drosophila* [61]. In all these studies the authors examine the genomes that have stable set of TEs. It was proposed that constraint on the efficiency of the piRNA machinery may be greater only at the first steps of a new TE invasion [17,21]. R strains with different reactivity serve as an excellent model to simulate such scenario and to explore the role of the piRNA factor polymorphism in the adaptive response to a new TE invasion. Indeed, variation in the production of primary piRNAs in R strains becomes apparent in the initial response to the *I*-element invasion. This does not affect active transposons because the mechanism of piRNA amplification, with participation of TE mRNAs, masks the differences in the amount of primary piRNAs (Fig 7). It is believed that TE insertions in the master loci provide adaptive immunity [6,7]. We develop this idea and suggest that such insertion could provide prompt and effective defense only if the efficiency of the primary piRNA production is sufficient.

Even though numerous genic piRNAs collinear to the cellular mRNAs are produced in different organisms, the influence of the piRNA production on the stability of mRNAs that serve as the piRNA precursors has not been explored. One hypothesis is that genic piRNAs production is a side-effect of the recognition of erroneous piRNA targets [28]. Nevertheless, the TJ protein encoded by the gene *tj* which harbors one of the major 3’ genic piRNA cluster, was shown to be upregulated in *piwi* mutant follicular cell clones, of late, but not of early egg chambers [35]. We describe two examples of the natural genic piRNA cluster polymorphisms and their influence on the level of mRNA that serve as the piRNA precursors. Different abundance of the *tj* 3‘UTR piRNAs in *Misy* and *Paris* strains does not significantly affect *tj* mRNA level whereas formation of the potent piRNA cluster within 3’UTR of gene *egl* leads to the decrease of the corresponding mRNA and protein. Steady-state mRNA level of genes whose 3’UTR generate piRNAs likely depends on the ratio between transcribed and piRNA-processed mRNA pools. Thus, production of 3’ genic piRNAs can slightly shift the abundance of corresponding mRNAs and proteins that may be evolutionarily significant.

The question arises: Is it advantageous or not for the genome to have strong primary piRNA expression? On the one hand, it provides efficient protection against *I*-element expansion, which might be considered a beneficial feature. Efficient production of 3’ genic piRNAs can alter the level of numerous gene transcripts and proteins which in the end could affect gene regulation network that may be evolutionarily significant. At the same time, a limited selective advantage for host transposon repression has been previously suggested [62]. Analysis of piRNA-mediated silencing has also revealed limits to the optimization of piRNA-mediated defense against active TEs [32]. We speculate that the necessity of telomere elongation by transpositions of telomeric retrotransposons explains this limitation; because, while enhanced production of piRNA can be advantageous for eradication of harmful TEs, it can also cause damage by targeting domesticated elements such as the telomeric retroelement *HeT-A*. Unexpectedly, genome-wide analysis revealed that *Paris*, which is resistant to *I*-element invasion, is also characterized by an extremely low copy number of the major telomeric retrotransposon *HeT-A*.

In the *Drosophila* germline, telomeric retrotransposon transcription and the rate of their transpositions onto the chromosome end are regulated by the piRNA pathway: The piRNA system suppresses excessive retrotransposon activity to maintain sufficient RNA levels to provide telomere elongation [51,63]. Telomeres represent an unusual sort of piRNA clusters, because they produce both piRNAs and their target mRNAs, which serve as templates for telomere elongation. *HeT-A* is particularly sensitive to piRNA pathway disruption, and demonstrates strong derepression in contrast to a modest response of *TART* [51,64]. We suggest that *HeT-A* might be also more sensitive than *TART* to the enhanced production of primary piRNAs. Only for *Paris*, we could connect strong suppression of the *I*-element to enhanced production of primary piRNAs in general. We speculate that exceptionally effective production of primary piRNAs in *Paris* could lead to a greater level of *HeT-A* piRNAs, which in the end would lead to elimination of *HeT-A* mRNA that are essential for telomere elongation (S15 Fig).

## Materials and methods

### *Drosophila* strains

R strains *w*^*K*^, *Misy*, *Paris*, *cn bw;e*, *cn;e*, and *Zola* were obtained from the collection of Institut de Genetique Humaine (CNRS), Montpellier, France. Reactivity was evaluated by measuring the percentage of non-hatching embryos laid by the progeny resulting from the cross of R females with *w*^*1118*^ males containing functional *I*-elements, according to a previously described procedure [12]. Strains bearing *spindle-E* (*spn-E*) mutations were *ru*^*1*^ *st*^*1*^ *spn-E*^*1*^ *e*^*1*^ *ca*^*1*^/*TM3*, *Sb*^*1*^ *e*^*s*^ and *ru*^*1*^ *st*^*1*^ *spn-E*^*hls3987*^ *e*^*1*^ *ca*^*1*^/*TM3*, *Sb*^*1*^ *e*^*s*^; *iso-1* (*y*^*1*^; *cn*^*1*^ *bw*^*1*^ *sp*^*1*^) is an isogenic strain used for whole-genome sequencing by the Drosophila Genome Project. The *Gaiano III (GIII)* strain carries the third chromosome with the *Tel* locus mutation affecting telomere length [46]. RAL-852 strain used in the analysis of *HeT-A* copy number was obtained from laboratory of Dr. T. Mackay, NCSU, Raleigh, NC.

### Northern analysis of small RNAs

Northern analysis of small RNAs was performed as previously described [4]. The *I*-element probe contained a fragment of *I*-element corresponding to nucleotides 2109–2481 of the GenBank entry M14954. The *HeT-A* antisense probe contained a fragment of the ORF containing nucleotides 4330–4690 of GenBank sequence DMU06920. A PCR fragment amplified using primers 5’-gatcatttccaagagcttttcct-3’ and 5’-taatacgactcactatagggagagggaataaatatcaacag-3’ was used for *traffic jam (tj)* antisense riboprobe preparation. *egl* probe was synthesized using a PCR fragment amplified with primers 5’-tacaattaatacgactcactataggcgacaaagatattaggaaacc-3 and 5’-cctaacaaacaaagcgcagacac-3’. Hybridization with P^32^ 5’-end-labelled oligonucleotide 5’-actcgtcaaaatggctgtgata-3’ complementary to miRNA-13b-1 was used as a loading control. The blots were visualized with the phosphorimager Typhoon FLA-9500 (Amersham).

### *In situ* RNA hybridization

*In situ* RNA analysis was carried out according to the previously described procedure [65] using a digoxigenin (DIG)-labeled strand-specific riboprobe and alkaline phosphatase (AP)-conjugated (diluted at 1/2000) anti-DIG antibodies (Roche). The 42AB-I probe corresponds to the genomic fragment chr2R:6261222–6261811 (BDGP assembly R6). The *I*-element probe contains a fragment of ORF2 corresponding to nucleotides 2109–2481 of the GenBank entry M14954. Fluorescence *in situ* hybridization (FISH) with polytene chromosomes was performed as described [66]. The *HeT-A* probe contains a fragment of *HeT-A* ORF, corresponding to 1746 to 4421 nucleotides in the GenBank sequence DMU 06920. The DNA probe was labeled using a DIG DNA labeling kit (Roche).

### RT-qPCR analysis

RNA was isolated from the ovaries of 3-day-old females obtained from young parents (F7) or from parents of mixed ages. cDNA was synthesized using random hexamer and SuperScriptII reverse transcriptase (Life Technologies). cDNA samples were analyzed by real-time quantitative PCR using SYTO-13 dye on LightCycler96 (Roche). Values were averaged and normalized to the expression level of the ribosomal protein gene *rp49*. Standard error of mean (SEM) for three independent RNA samples was calculated. The primers used are listed in S4 Table.

### Chromatin immunoprecipitation

~150 pairs of 3-day-old female ovaries obtained from young parents (F7) were dissected for every IP experiment. ChIP was performed according to the published procedure [67]. 2.5 μg and 50 ng of chromatin were taken for each ChIP and input probe, respectively. Protein G Agarose/Salmon sperm DNA (Millipore) was used without preincubation with chromatin. Chromatin was immunoprecipitated with rat Rhi antiserum [68]. Quantitative PCR was conducted with Lightcycler96 (Roche). Percent input was calculated using the formula: % input = (2^(Ct input–Ct IP)) × *F*_*d*_ × 100, where % input is the ChIP efficiency expressed in percent when compared with total DNA; Ct IP and Ct input are threshold cycles for ChIP and input samples, respectively; *F*_*d*_ is the dilution factor. SEM of triplicate PCR measurements for two biological replicates was calculated.

### Small RNA libraries

Small RNAs 19–29 nt in size from total ovarian RNA isolated from *cn bw;e*, *Paris*, *Misy*, and *Zola* strains and from 0-2-hour-old embryos of *Paris* and *Misy* strains were cloned as previously described [69]. Libraries were barcoded according to the Illumina TrueSeq Small RNA sample prep kit manual and sequenced using the Illumina HiSeq-2000 system. After clipping the Illumina 3’-adapter sequence, small RNA reads that passed a quality control and minimal length filter (>18nt) were mapped (allowing 0 mismatches) to the *Drosophila melanogaster* genome (Apr. 2006, BDGP assembly R5/dm3) using bowtie [70]. In order to identify piRNAs, the sequenced small RNAs were mapped to the canonical sequences of transposable elements (http://www.fruitfly.org/p_disrupt/TE.html) with the allowance of up to 3 mismatches. Small RNA libraries were normalized to library depth. The plotting of size distributions, read coverage, and nucleotide biases were performed as described previously [4]. Ovarian and embryonic small RNA-seq data for *cn bw;e*, *Zola*, *Paris*, *and Misy* strains were deposited at Gene Expression Omnibus (GEO), accession number GSE83316. Small RNA-seq data from ovaries of *w*^*K*^ (GSM1024091) and *iso-1* (GSM1123781) strains were described previously [4,5]. Small RNA-seq data from ovaries of 16 RAL strains from the DGRP project [37] were obtained from NCBI SRA, SRP019948 [38].

### Genome sequencing

Paired-end (~250 bp fragment size) and mate-pair (~8 kb fragment size) libraries from *Paris* and *Misy* strain genomic DNA were prepared according to the Illumina standard protocols and sequenced on the Illumina HiSeq 2000. The genomic deep sequencing data were deposited in the NCBI SRA Database, SRP076499. DNA-seq data of *w*^*K*^ (SRA accession number SRP021106) were described previously [5]. Insertion sites of *I*-elements in *Paris*, *Misy*, and *w*^*K*^ genomes were identified by using the *mcclintock* meta-pipeline (https://github.com/bergmanlab/mcclintock) with the canonical sequences of TEs (http://www.fruitfly.org/p_disrupt/TE.html) and the annotated TE insertion sites in the reference *iso-1* strain from FlyBase (BDGP r.6). Only the non-redundant unambiguous TE insertions were taken into account.

## Supporting information

S1 File Supporting materials and methods. This file contains information about antibodies used in the study, details on the DNA-seq and RNA-Seq data statistical analysis and references.
(PDF)

S1 Fig Northern analysis of the RNA isolated from the ovaries of *iso-1*, *Misy*, *Paris* and RAL-852 strains. Hybridization was done with *I*-element riboprobe to detect antisense piRNAs. Lower panel represents hybridization to *mir-13b1* microRNA. P^32^-labeled RNA oligonucleotides were used as size markers.
(TIF)

S2 Fig (A) Negative correlation between reactivity and content of single-mapped *I*-element specific piRNAs from *42AB* piRNA cluster for R strains. Spearman correlation tests: r = -0.90, P-value <0.1. The line depicts the results of linear regression analysis for the level of reactivity and amount of piRNAs. R^2^—adjusted squared R (P-value < 0.1); the grey zone illustrates the 90% confidence interval. (B) *I*-element fragments located within *42AB* are intact in R strains. Dot plots show pairwise sequence alignments in *iso-1*, *Paris*, *Misy*, *Zola and cn bw*,*e* strains; percent identity is indicated. PCR analysis of genomic DNA showed that *42AB-I*-element region of the *Zola* strain contains full-length retrotransposon insertion *Bs2* (*Jockey* family), which is also present in the genome of the *iso-1*, while *Paris*, *Misy* and *cn bw; e* strains do not contain this insertion. The presence/absence of the *Bs2* insertion does not correlate with reactivity. Primers used for PCR of genomic DNA and sequencing are indicated by arrowheads and arrows, respectively. PCR fragments amplified with 1R and 3R primers (*Misy*, *Paris* and *cn bw; e* strains) or 3F and 3R primers (*Zola* strain) were sequenced using 3R and 4R primers. Sequence similarity indicates that this region is conserved.
(TIF)

S3 Fig piRNA production of *I*-element fragments located within piRNA clusters. (A) Single-mapped small RNAs specific to the *I*-element fragments within piRNA clusters 134, 75, and 76 in *Misy*, *Paris*, *Zola* and I strain *iso-1*. Mapping of small RNA reads was done to the reference genome. Sequencing of *I*-element-containing regions in R strains (S3B Fig) revealed minor sequence variations that were not considered in alignments. Red and grey boxes correspond to the *I*-element or other TE fragments, respectively; dashed lines indicate matching of the *I*-element fragments located within piRNA clusters to the canonical copy. Reads mapped to the sense strand are shown in blue, and antisense in brown. (B) The presence of the *I*-element fragments within clusters 134, 75, and 76 in *Misy* and *Paris* genomes has been confirmed by PCR of genomic DNA followed by sequencing. Dot plots show pairwise sequence alignments with percent identity from clusters 134, 75, 76 in *iso-1*, *Paris* and *Misy* strains. Primers used for PCR of genomic DNA and sequencing are indicated. *Rt1b* insertion was identified within *I*-element fragment located in cluster 134 in *Paris* strain.
(TIF)

S4 Fig Transcription of different regions of the piRNA cluster at *42AB* in R strains. (A) RT-qPCR analysis of *42AB* piRNA cluster (*I*-element region) expression in ovaries of 3-day-old R strain females obtained from parents of mixed ages. Relative RNA steady-state level is shown. (B) RT-qPCR analysis of the expression levels of two regions of the *42AB* cluster (42AB-1 and 42AB-2), harboring fragments of active TEs. Asterisks indicate statistically significant differences relative to *Misy* (* P < 0.05 to 0.01, ** P < 0.01 to 0.001, t-test).
(TIF)

S5 Fig Strain-specific ancestral *I*-element insertions (addition to S2 Table). piRNA production in the regions comprising predicted *I*-element insertions in *Paris* (A, B) and *Misy* (C) strains. Single-mapped small RNAs are shown. Reads mapped to the sense strand are shown in blue, and antisense in brown. Black and grey boxes correspond to the TE fragments annotated in the genome of *iso-1* strain. Arrows indicate sites or interval of predicted *I*-element insertions.
(TIF)

S6 Fig Scatter plot shows normalized TE-specific piRNAs (in TPM, 24–29 nt reads were considered) in 0-2-hour-old embryos in *Paris* and *Misy* strains. The piRNA expression was additionally normalized to the log_2_-transformed and RPM-normalized genomic abundance of the corresponding TEs. The color of dots indicates the type of TEs according to its capacity to maternal deposition in embryos according to [24].
(TIF)

S7 Fig Comparison of small RNA populations in ovaries of R strains. (A) The estimation of the abundance of different classes of small RNAs in R strains. The number of 21 nt and 24–29 nt reads mapping to the piRNA clusters [1] (single-mapped reads are considered), endo-siRNA generated loci [71], and canonical TE copies were normalized (RPM) and log_2_-transformed. (B) The distribution of Spearman correlation coefficients of piRNA ping-pong profiles of germinal TEs in R and *iso-1* stains. The coefficients of the correlation of z-normalized ping-pong profiles were determined for each individual TE in the given R strain and in the *iso-1* strain. A comparison of the distribution of Spearman correlation coefficients did not revealed a significant difference between R strains (for Paris/Misy p = 0.89, for Paris/w^K^ p = 0.73; Wilcoxon test).
(TIF)

S8 Fig Uni-strand piRNA clusters produce more piRNAs in the *Paris* than in *w*^*K*^ strong R strain (addition to Fig 4). (A) Scatter plot of log_2_-transformed and RPM-normalized small RNA expression in ovaries of *Paris* and *w*^*K*^ strains. piRNA expression was additionally normalized to the log_2_-transformed and RPM-normalized genomic abundance of the corresponding TEs. The color of dots indicates the type of TEs according to its capacity for maternal deposition in embryos according to [24]. Dashed lines depict the results of linear regression analysis for TEs with high maternal deposition (genuine germinal TEs, red) and for TEs with weak maternal deposition (genuine somatic TEs, blue). R^2^—adjusted squared R (P-value < 0.1). (B) Relative abundance (*Paris/ w*^*K*^) of a normalized number of single-mapped small RNA reads (RPM, 24–29 nt reads were considered) mapping to TE copies located within uni-strand piRNA clusters #8 *(flamenco*, expressed in follicular cells) and #2 (*20A*, expressed in the germline).
(TIF)

S9 Fig Production of genic piRNAs in R strains (addition to Fig 5). (A) Northern analysis of the small RNAs isolated from the ovaries of *Misy*, *cn bw;e*, *Zola*, *Paris* strains. Hybridization was done with the *tj* and *egl* antisense riboprobes. The lower panel represents hybridization to the *mir-13b1* microRNA. (B) The number of 24–29 nt reads mapping to the six most productive genic piRNA clusters were normalized (RPM) and log_2_-transformed. (C) A comparison of genome sequences revealed no significant differences in the regions comprising six major genic piRNA clusters in *Paris*, *Misy* and *w*^*K*^ strains. Dot plots show pairwise sequence alignments in *iso-1*, *Paris*, *Misy*, and *w*^*K*^ strains; percent identity is indicated.
(TIF)

S10 Fig Clustering and heatmap analysis for small RNA data shows fold changes (log_2_) of normalized read values (RPM) corresponding to the most potent 3’ genic piRNA clusters in follicular cells [35], in *Paris*, *Misy*, and 16 RAL strains from the DGRP [37,38]. (TIF)

S11 Fig Western blot of *Misy* and *Paris* ovary extracts probed with antibodies against pi- and siRNA pathway proteins. Antibodies used in the study are described in S1 File, Supporting Materials and Methods.
(TIF)

S12 Fig *HeT-A* copy number is dramatically decreased in the telomeres of the *Paris* strain. qPCR on the genomic DNA of indicated strains was done to estimate normalized (to the single-copy *rp49* gene) *HeT-A* copy number relative to the reference strain *iso-1*. Two *Paris* sublines independently handled in different laboratories for several years, *Paris*^*R*^ and *Paris*^*F*^, showed identical low *HeT-A* copy number. RAL-852 was chosen as a strain having the lowest *HeT-A* copy number in the DGRP collection [48].
(TIF)

S13 Fig DNA FISH with telomeric retroelement *HeT-A* probe on polytene chromosomes of salivary glands from *Paris*, *Misy*, and RAL-852 *D*. *melanogaster* strains. FISH analysis detected hybridization signals at some telomeres of *Misy* and RAL-852 strains. No hybridization signals were detected at the telomeres of the *Paris* strain. *HeT-A* is in red. Chromosomes are stained with DAPI (gray).
(TIF)

S14 Fig Genomic occupancy and small RNA production of telomeric retrotransposons in R strains (addition to Fig 6) (A) Telomeric retrotransposon *TAHRE*, *TART-A*, *TART-B* and *TART-C* genomic sequence coverage in R strains. Analysis of paired-end genomic DNA libraries. (B) The density of small RNAs in ovaries along the *TART-A*, *TART-B* and *TART-C* canonical sequences is shown. Length distribution of *TART* small RNAs is plotted on the right. Percentages of reads having 1U bias are indicated for each strand (only 24–29 nt reads were considered).
(TIF)

S15 Fig A model for the role of the piRNA system in the regulation of telomere length. Telomeric repeats produce both piRNAs and their target mRNAs, which serve as templates for telomere elongation. piRNA elimination as a result of piRNA-pathway mutations causes telomere elongation [51]. An increased level of piRNA production as a result of the piRNA component polymorphism could cause an enhanced level of piRNAs specific to the main telomeric element *HeT-A* and telomere shortening.
(TIF)

S1 Table Differential expression analysis of the abundance of TE-specific small RNAs in strong (*Misy* and *w*^*K*^) versus weak (*Zola* and *Paris*) R-strains. (XLS)

S2 Table Identification of ancestral *I*-element insertions in genomes of *Paris*, *Misy*, and *w*^*K*^ *D*. *melanogaster* R strains. (XLS)

S3 Table piRNA factor polymorphism in *Paris*, *Misy*, and RAL *D*. *melanogaster* strains. (XLS)

S4 Table Primers used in the study. (XLS)
