# Supplementary material for: Natural variation of piRNA expression affects immunity to transposable elements
Source: PLoS Genet. 2017 Apr 27;13(4):e1006731. doi: 10.1371/journal.pgen.1006731 (PMC5407775; doi:10.1371/journal.pgen.1006731)
Supplement: S1 File — This file contains information about antibodies used in the study, details on the DNA-seq and RNA-Seq data statistical analysis and references. (PDF) [file pgen.1006731.s001.pdf]

Materials and Methods

Antibodies

To generate anti-Aub and anti-SpnE antibodies, PCR fragments encoding 1-331 aminoacids of Aub or 1043-1420 aminoacids of SpnE were cloned into the pET28b vector. Expression was carried out in *Escherichia coli* strain Rosetta (DE3) (Novagen). Expressed proteins were purified according to the manufacturer's protocol under denaturing conditions using a 1-mL chelating HiTrap column (GE Healthcare) charged with Ni^{2+}. Recombinant proteins were used for the generation of rat polyclonal antibodies by the Immunochemistry laboratory (Branch of IBC RAS, Pushchino, Russia).

Following antibodies were used in Western blotting:
anti-SpnE, rat polyclonal antiserum;
anti-Aub, rat polyclonal antiserum;
ant-Armi, murine monoclonal antibodies, kindly provided by M. Siomi [1];
anti-Rhino, rat antiserum [2];
anti-Ago2, Abcam, #ab5072;
anti-Ago3, murine monoclonal anti-AGO3 9G3 antibody, kindly provided by M. Siomi [3];
anti-Squash, Squash 1F3-1B10, DSHB;
anti-PIWI, rabbit polyclonal antibodies, kindly provided by G. Hannon [4];
anti-Egl, rabbit polyclonal, kindly provided by R. Lehmann [5];
anti-α-tubulin, Sigma-Aldrich, T9026.

Analysis of small RNA data

The differential expression of piRNAs corresponding to the TE consensus sequencies (http://www.fruitfly.org/p_disrupt/TE.html) was performed by using the *edgeR* package (v. 3.16.5) [6] from R/Bioconductor (v. 3.3.2) [7]. The pairs of strong (*Misy* and w^K) and weak (*Paris* and *Zola*) R-strains were used as pseudo-replicates. FDR < 0.1 was chosen as a cutoff value.

To estimate the abundance of siRNAs (21 nt), miRNAs (21-23 nt) and piRNAs (24-29 nt) in ovaries of R strains, the number of reads uniquely mapped to the endo-siRNA generated loci (ESI-siRNA, [8]), annotated miRNAs genes (miRBase, v. 20), piRNA clusters (ML-piRNA and ML-siRNA, [4]) and consensuses of TEs (TE-piRNA, TE-siRNA) were counted, RPM-normalized.

The sequences of the longest 3'UTRs of known genic piRNA clusters [9] were extracted by using *TxDb* (version 3.2.2) and *GenomicFeatures* (version 1.24.5) packages of R/Biocoductor software [10]. The number of reads uniquely mapped to the retrieved 3'UTRs in strains R and RAL were RPM-normalized and used to create heatmaps. Nonparametric bootstrapping procedure for validation the strains' clustering was performed by using *clusterboot* method (clustermethod=hclustCBI, method="ward", B=1000, bootmethod="boot") implemented in *fpc* package (version 2.1-10) of R software.

The ping-pong signature was detected according to [11]. The global comparison of the piRNA ping-pong profiles in R strains with strain *iso-1* was done by determination of the individual Spearman correlation coefficients of z-scored ping-pong signature for each TE family. The obtained correlation coefficients were used for the construction of boxplots and testing the significance of difference of ping-pong profiles between R strains by Wilcoxon rank sum test.

To compare small RNAseq from ovaries and embryos we calculated TPM (transcripts per kilobase per million) values as the most stable unit used for comparison of expression across different samples.

Analysis of DNA-seq data

To compare the copy number of TEs in genomes of R-strains the reads from paired-end and mate-paired DNA-seq libraries after the removing of the adapter sequences were trimmed up to 50 nt and collapsed. Then the files containing reverse reads of paired-end or mate-pair libraries were reverse complemented and combined with the forward reads and the resulted reads were aligned to the canonical sequences of TEs (http://www.fruitfly.org/p_disrupt/TE.html) by bowtie with allowing of up to 3 mismatches. To construct the coverage of TEs by DNA-seq reads, the number of DNA-seq reads were counted in 100 bp bin intervals with 200 bp smoothing windows; the obtained RPM-normalized profiles were additionally smoothed by applying the cubic spline interpolation. To statistically test the differences of global TE copy abundances between genomes of R-strains or between paired-end and mate-pair datasets, the number of RPM-normalized and log_2-transformed reads in bins were compared by the using the two-sided Wilcoxon rank sum test. The P-value = 10^{-5} was chosen as the cut-off value.

The assembled genomic sequences of RAL strains from the DGRP project [12] were retrieved from the Drosophila Genome Nexus web-site (http://www.johnpool.net/genomes.html). The assembling of draft genomes of *Paris* and *Misy* strains was performed with mate-pair DNA-seq data by *velvet* assembler (-k 64) [13]. The N50s of the resulted contigs were 10738 bp for the *Paris* strain and 35726 bp for the *Misy* strain. The allelic variants were identified with Scipio

software [14] by querying the largest isoform of piRNA pathway factors obtained from UniProt against assembled genomes of RAL and R strains.

The sequences of *I*-element fragments within piRNA clusters were determined by sequencing of PCR products obtained with cluster-specific primers (S4 Table, S2 and S3 Fig). To obtain the genomic sequences of 3'UTR piRNA clusters, paired-end reads were mapped to *dm3* genome by using *bwa* [15] followed by the removal of duplicates, indel realignment and quality score recalibration of aligned reads using the GATK software package [16]. The extraction of consensus genomic sequences from read pileups was performed by applying *samtools* [17] and *bcftools* software packages.The genomic sequence alignments of 3’UTR piRNA clusters and piRNA cluster fragments containing *I*-elements in *Paris*, *Misy, Zola* and *cn bw; e* strains was performed by using the MUSCLE software [18] and visualized as dotplots by using *dotPlot* function of *seqinr* R package (wsize = 5, wstep = 5, nmatch = 5) (v. 3.3.3) [10,19].

References

1. Saito K, Ishizu H, Komai M, Kotani H, Kawamura Y, et al. (2010) Roles for the Yb body components Armitage and Yb in primary piRNA biogenesis in Drosophila. Genes Dev 24: 2493-2498.
2. Radion E, Ryazansky S, Akulenko N, Rozovsky Y, Kwon D, et al. (2016) Telomeric Retrotransposon HeT-A Contains a Bidirectional Promoter that Initiates Divergent Transcription of piRNA Precursors in Drosophila Germline. J Mol Biol.
3. Gunawardane LS, Saito K, Nishida KM, Miyoshi K, Kawamura Y, et al. (2007) A slicer-mediated mechanism for repeat-associated siRNA 5' end formation in Drosophila. Science 315: 1587-1590.
4. Brennecke J, Aravin AA, Stark A, Dus M, Kellis M, et al. (2007) Discrete small RNA-generating loci as master regulators of transposon activity in Drosophila. Cell 128: 1089-1103.
5. Mach JM, Lehmann R (1997) An Egalitarian-BicaudalD complex is essential for oocyte specification and axis determination in Drosophila. Genes Dev 11: 423-435.
6. Robinson MD, McCarthy DJ, Smyth GK (2010) edgeR: a Bioconductor package for differential expression analysis of digital gene expression data. Bioinformatics 26: 139-140.
7. Huber W, Carey V, Davis S, Hansen KD, Morgan M (2015) The Bioconductor channel in F1000Research. F1000Res 4: 217.
8. Czech B, Malone CD, Zhou R, Stark A, Schlingeheyde C, et al. (2008) An endogenous small interfering RNA pathway in Drosophila. Nature 453: 798-802.
9. Robine N, Lau NC, Balla S, Jin Z, Okamura K, et al. (2009) A broadly conserved pathway generates 3'UTR-directed primary piRNAs. Curr Biol 19: 2066-2076.
10. Huber W, Carey VJ, Gentleman R, Anders S, Carlson M, et al. (2015) Orchestrating high-throughput genomic analysis with Bioconductor. Nat Methods 12: 115-121.
11. Antoniewski C (2014) Computing siRNA and piRNA overlap signatures. Methods Mol Biol 1173: 135-146.
12. Mackay TF, Richards S, Stone EA, Barbadilla A, Ayroles JF, et al. (2012) The Drosophila melanogaster Genetic Reference Panel. Nature 482: 173-178.

13. Zerbino DR, Birney E (2008) Velvet: algorithms for de novo short read assembly using de Bruijn graphs. Genome Res 18: 821-829.
14. Keller O, Odronitz F, Stanke M, Kollmar M, Waack S (2008) Scipio: using protein sequences to determine the precise exon/intron structures of genes and their orthologs in closely related species. BMC Bioinformatics 9: 278.
15. Li H, Durbin R (2010) Fast and accurate long-read alignment with Burrows-Wheeler transform. Bioinformatics 26: 589-595.
16. McKenna A, Hanna M, Banks E, Sivachenko A, Cibulskis K, et al. (2010) The Genome Analysis Toolkit: a MapReduce framework for analyzing next-generation DNA sequencing data. Genome Res 20: 1297-1303.
17. Li H, Handsaker B, Wysoker A, Fennell T, Ruan J, et al. (2009) The Sequence Alignment/Map format and SAMtools. Bioinformatics 25: 2078-2079.
18. Edgar RC (2004) MUSCLE: multiple sequence alignment with high accuracy and high throughput. Nucleic Acids Res 32: 1792-1797.
19. Charif D, Lobry JR (2007) SeqinR 1.0-2: a contributed package to the R project for statistical computing devoted to biological sequences retrieval and analysis. In: Bastolla U, Porto M, Roman HE, Vendruscolo M, editors. Structural Approaches to Sequence Evolution: Molecules, Networks, Populations, Biological and Medical Physics, Biomedical Engineering. New York: Springer Verlag. pp. 207–232.
