# Supplementary figures and images for: Natural variation of piRNA expression affects immunity to transposable elements
Source: PLoS Genet. 2017 Apr 27;13(4):e1006731. doi: 10.1371/journal.pgen.1006731 (PMC5407775; doi:10.1371/journal.pgen.1006731)

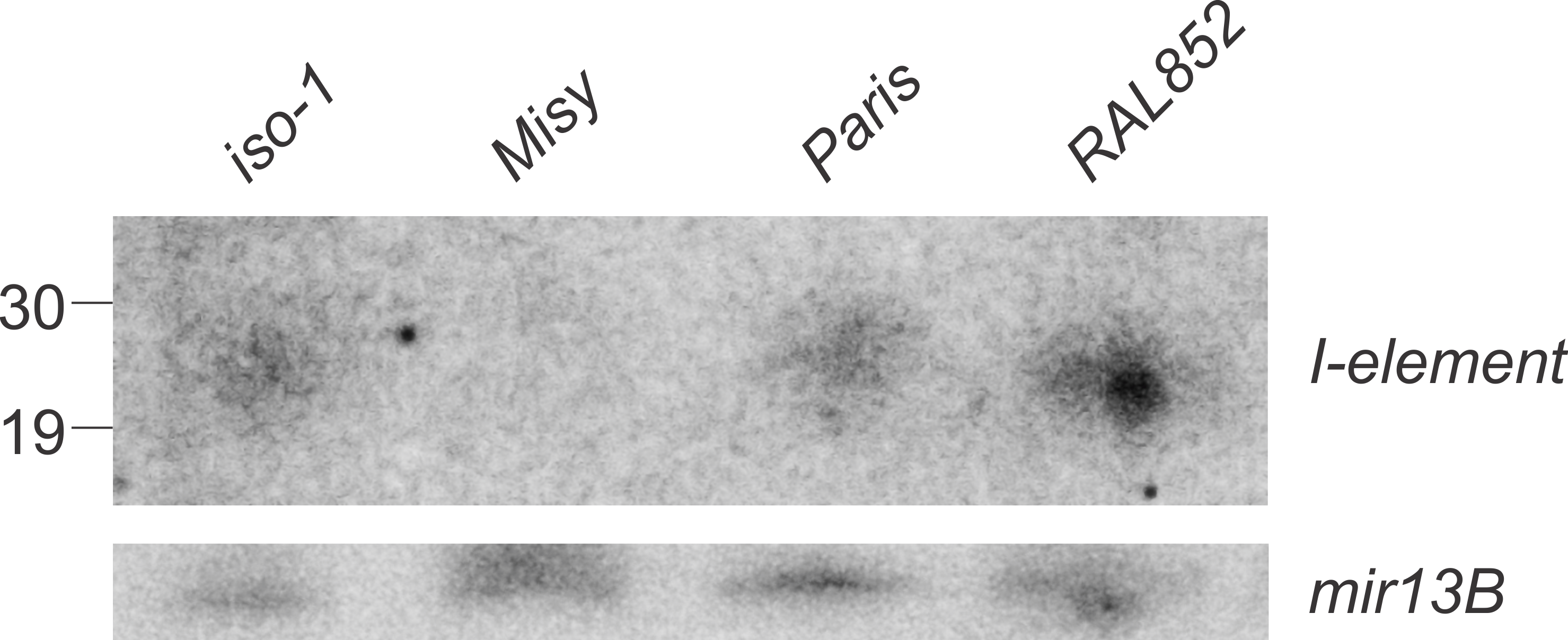

Supplement: S1 Fig — Hybridization was done with I-element riboprobe to detect antisense piRNAs. Lower panel represents hybridization to mir-13b1 microRNA. P32-labeled RNA oligonucleotides were used as size markers. (TIF) [file pgen.1006731.s002.tif]

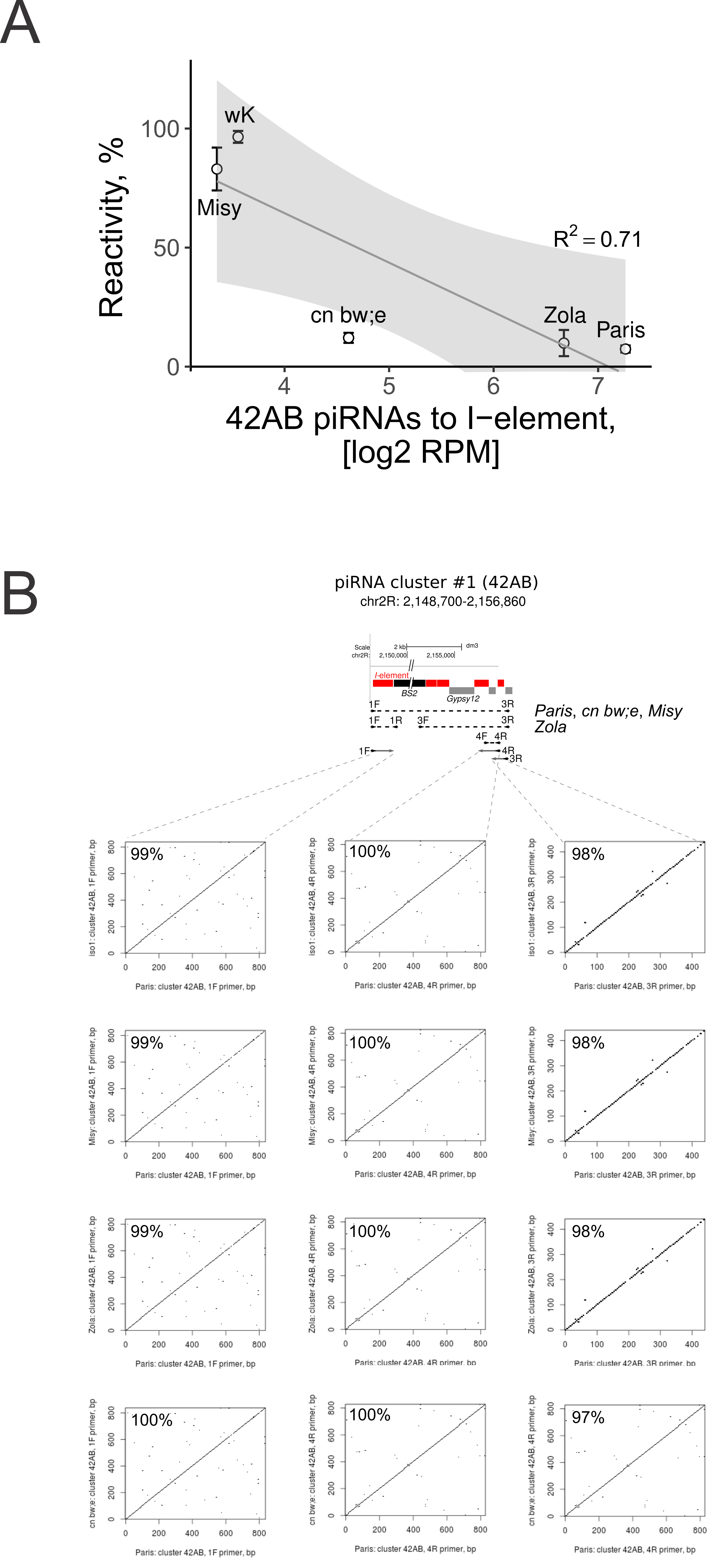

Supplement: S2 Fig — (A) Negative correlation between reactivity and content of single-mapped I-element specific piRNAs from 42AB piRNA cluster for R strains. Spearman correlation tests: r = -0.90, P-value <0.1. The line depicts the results of linear regression analysis for the level of reactivity and amount of piRNAs. R2—adjusted squared R (P-value < 0.1); the grey zone illustrates the 90% confidence interval. (B) I-element fragments located within 42AB are intact in R strains. Dot plots show pairwise sequence alignments in iso-1, Paris, Misy, Zola and cn bw,e strains; percent identity is indicated. PCR analysis of genomic DNA showed that 42AB-I-element region of the Zola strain contains full-length retrotransposon insertion Bs2 (Jockey family), which is also present in the genome of the iso-1, while Paris, Misy and cn bw; e strains do not contain this insertion. The presence/absence of the Bs2 insertion does not correlate with reactivity. Primers used for PCR of genomic DNA and sequencing are indicated by arrowheads and arrows, respectively. PCR fragments amplified with 1R and 3R primers (Misy, Paris and cn bw; e strains) or 3F and 3R primers (Zola strain) were sequenced using 3R and 4R primers. Sequence similarity indicates that this region is conserved. (TIF) [file pgen.1006731.s003.tif]

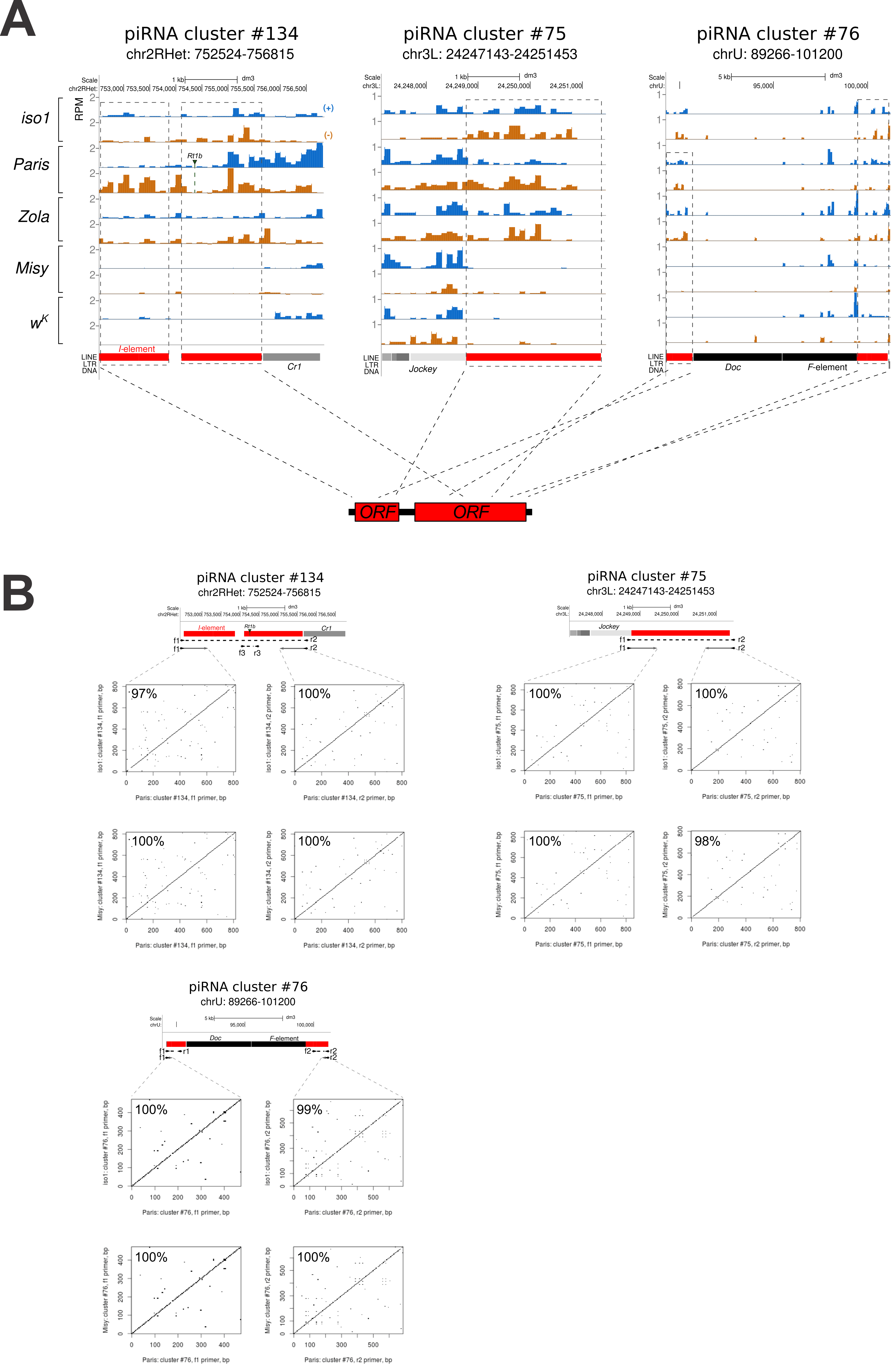

Supplement: S3 Fig — (A) Single-mapped small RNAs specific to the I-element fragments within piRNA clusters 134, 75, and 76 in Misy, Paris, Zola and I strain iso-1. Mapping of small RNA reads was done to the reference genome. Sequencing of I-element-containing regions in R strains (S3B Fig) revealed minor sequence variations that were not considered in alignments. Red and grey boxes correspond to the I-element or other TE fragments, respectively; dashed lines indicate matching of the I-element fragments located within piRNA clusters to the canonical copy. Reads mapped to the sense strand are shown in blue, and antisense in brown. (B) The presence of the I-element fragments within clusters 134, 75, and 76 in Misy and Paris genomes has been confirmed by PCR of genomic DNA followed by sequencing. Dot plots show pairwise sequence alignments with percent identity from clusters 134, 75, 76 in iso-1, Paris and Misy strains. Primers used for PCR of genomic DNA and sequencing are indicated. Rt1b insertion was identified within I-element fragment located in cluster 134 in Paris strain. (TIF) [file pgen.1006731.s004.tif]

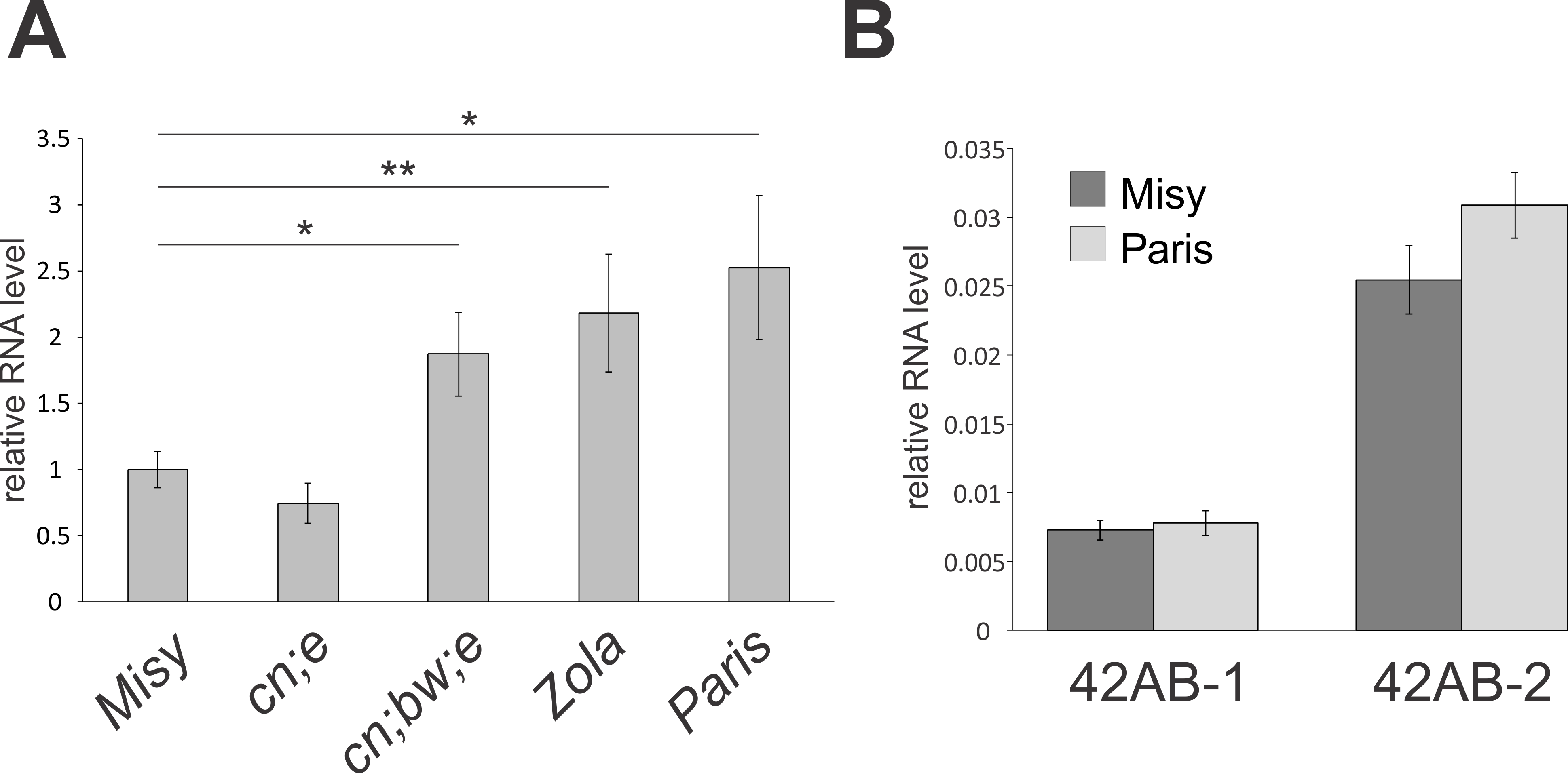

Supplement: S4 Fig — (A) RT-qPCR analysis of 42AB piRNA cluster (I-element region) expression in ovaries of 3-day-old R strain females obtained from parents of mixed ages. Relative RNA steady-state level is shown. (B) RT-qPCR analysis of the expression levels of two regions of the 42AB cluster (42AB-1 and 42AB-2), harboring fragments of active TEs. Asterisks indicate statistically significant differences relative to Misy (* P < 0.05 to 0.01, ** P < 0.01 to 0.001, t-test). (TIF) [file pgen.1006731.s005.tif]

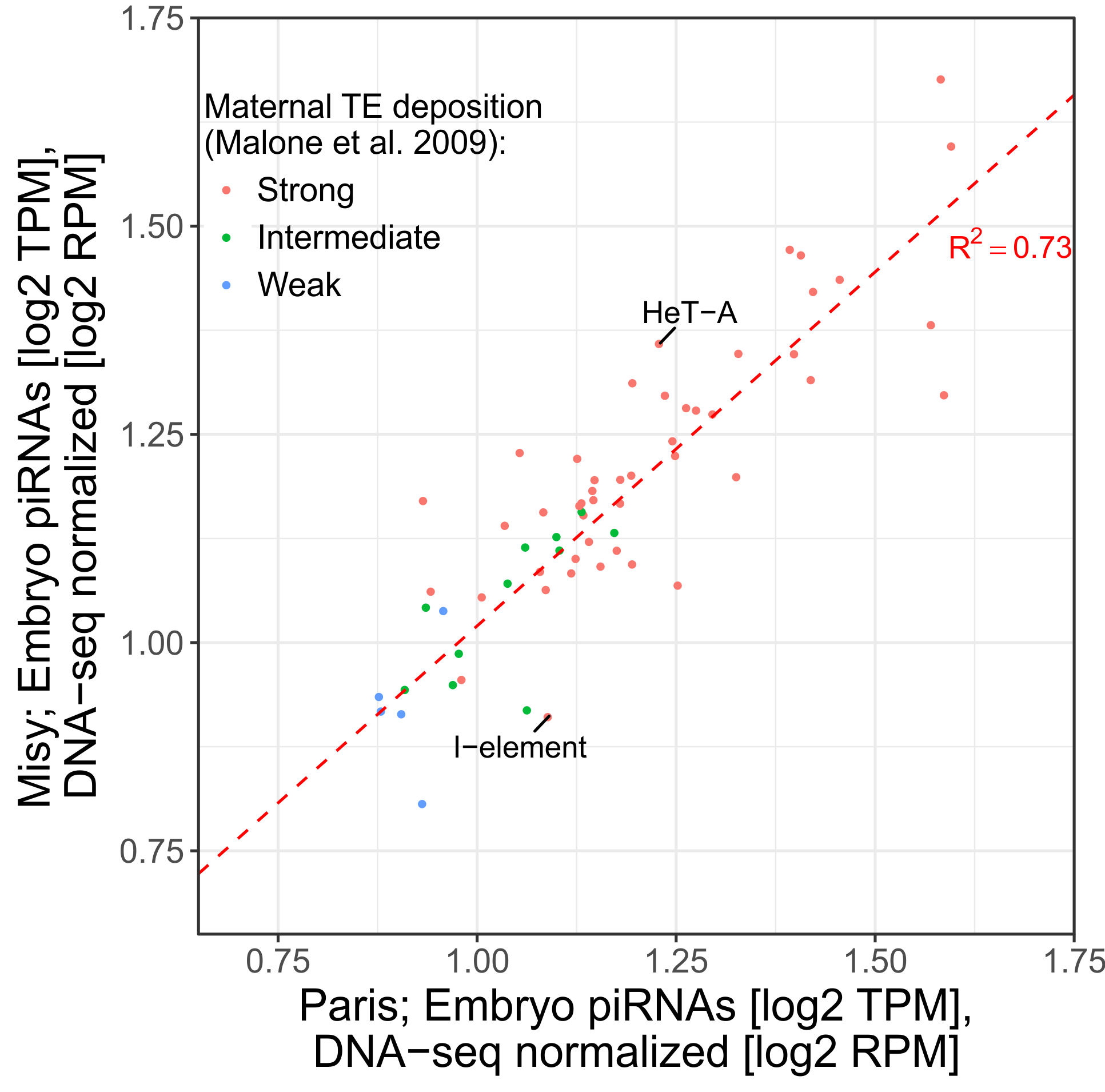

Supplement: S6 Fig — The piRNA expression was additionally normalized to the log2-transformed and RPM-normalized genomic abundance of the corresponding TEs. The color of dots indicates the type of TEs according to its capacity to maternal deposition in embryos according to [24]. (TIF) [file pgen.1006731.s007.tif]

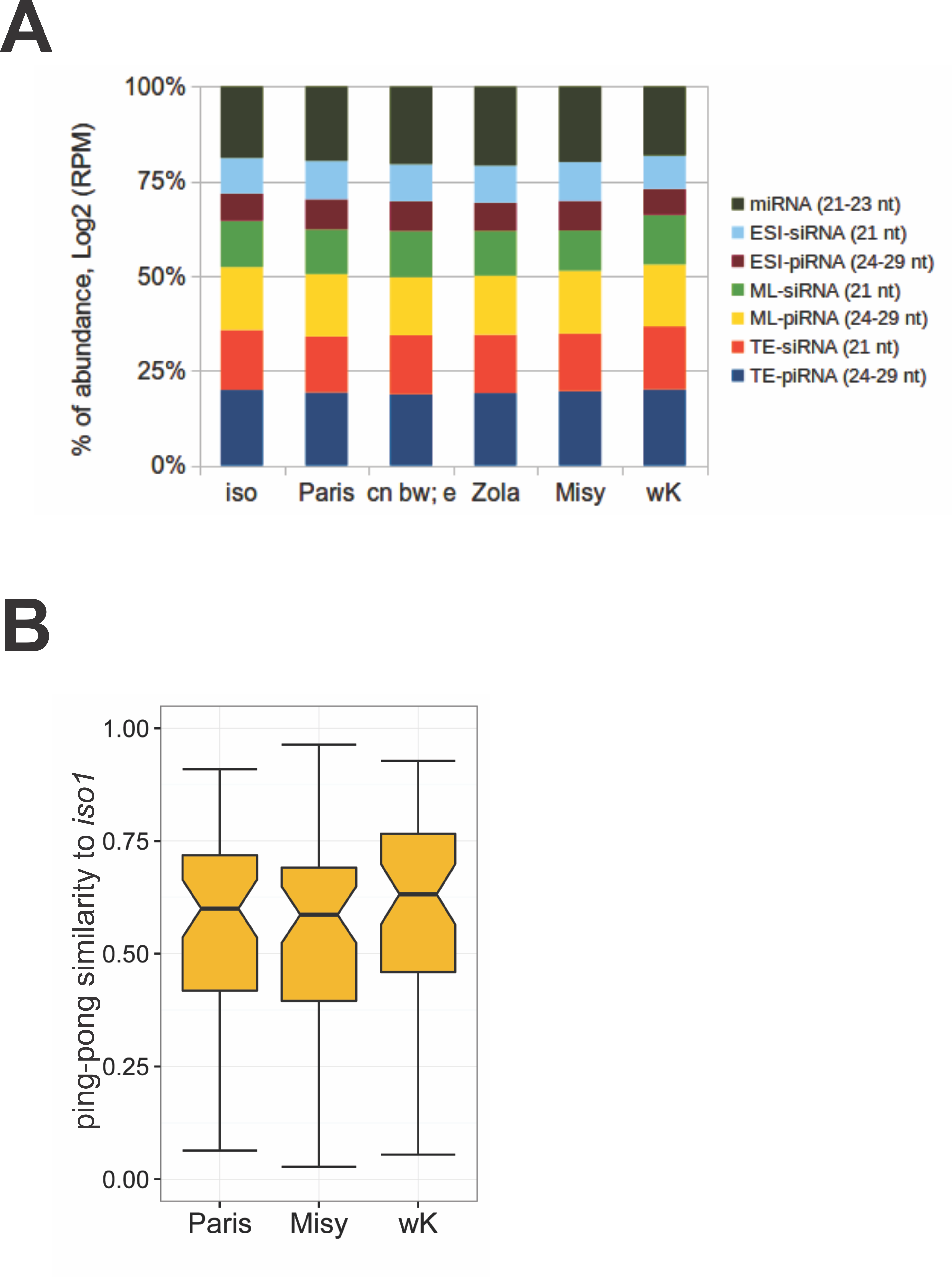

Supplement: S7 Fig — (A) The estimation of the abundance of different classes of small RNAs in R strains. The number of 21 nt and 24–29 nt reads mapping to the piRNA clusters [1] (single-mapped reads are considered), endo-siRNA generated loci [71], and canonical TE copies were normalized (RPM) and log2-transformed. (B) The distribution of Spearman correlation coefficients of piRNA ping-pong profiles of germinal TEs in R and iso-1 stains. The coefficients of the correlation of z-normalized ping-pong profiles were determined for each individual TE in the given R strain and in the iso-1 strain. A comparison of the distribution of Spearman correlation coefficients did not revealed a significant difference between R strains (for Paris/Misy p = 0.89, for Paris/wK p = 0.73; Wilcoxon test). (TIF) [file pgen.1006731.s008.tif]

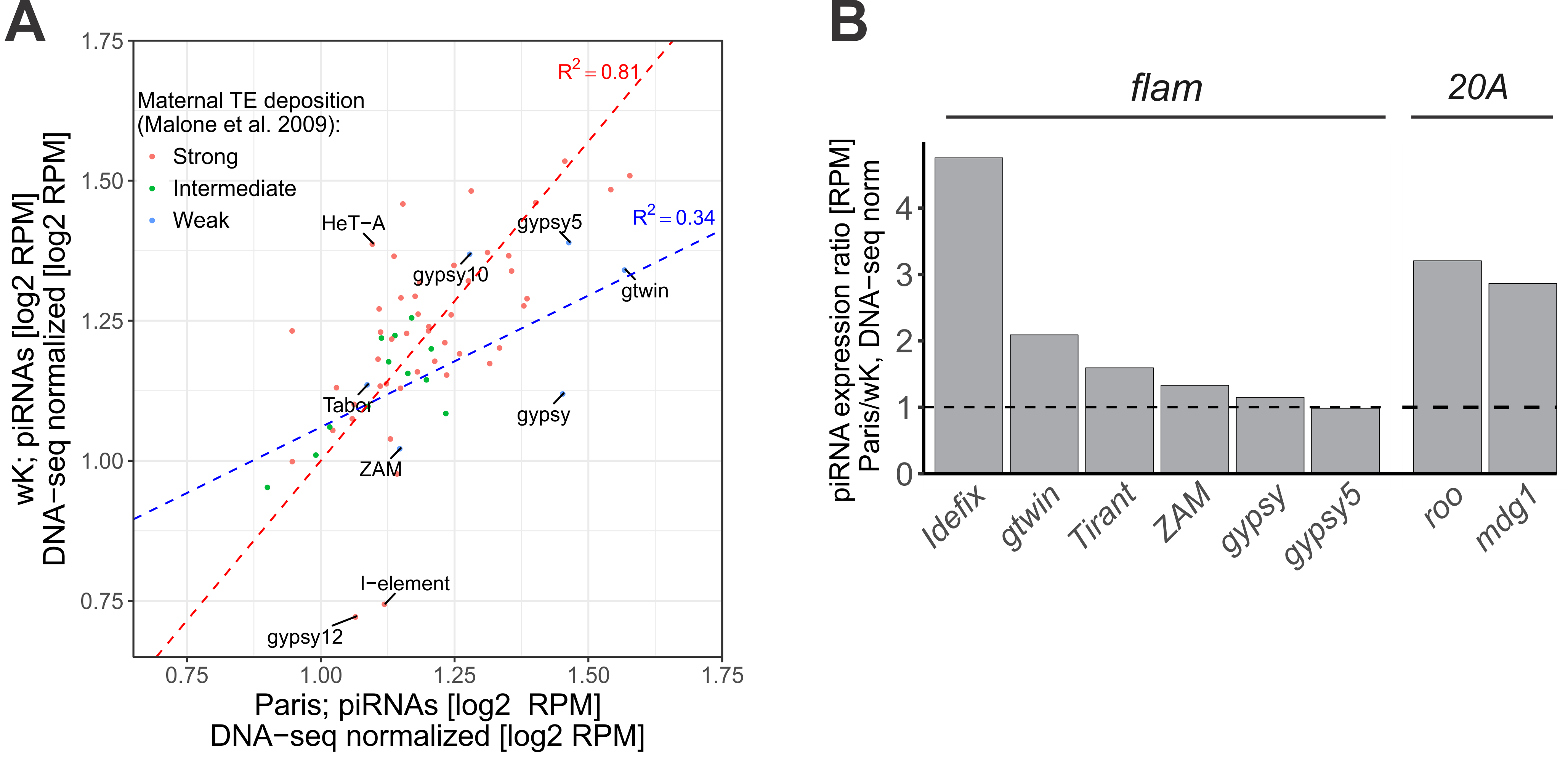

Supplement: S8 Fig — (A) Scatter plot of log2-transformed and RPM-normalized small RNA expression in ovaries of Paris and wK strains. piRNA expression was additionally normalized to the log2-transformed and RPM-normalized genomic abundance of the corresponding TEs. The color of dots indicates the type of TEs according to its capacity for maternal deposition in embryos according to [24]. Dashed lines depict the results of linear regression analysis for TEs with high maternal deposition (genuine germinal TEs, red) and for TEs with weak maternal deposition (genuine somatic TEs, blue). R2—adjusted squared R (P-value < 0.1). (B) Relative abundance (Paris/ wK) of a normalized number of single-mapped small RNA reads (RPM, 24–29 nt reads were considered) mapping to TE copies located within uni-strand piRNA clusters #8 (flamenco, expressed in follicular cells) and #2 (20A, expressed in the germline). (TIF) [file pgen.1006731.s009.tif]

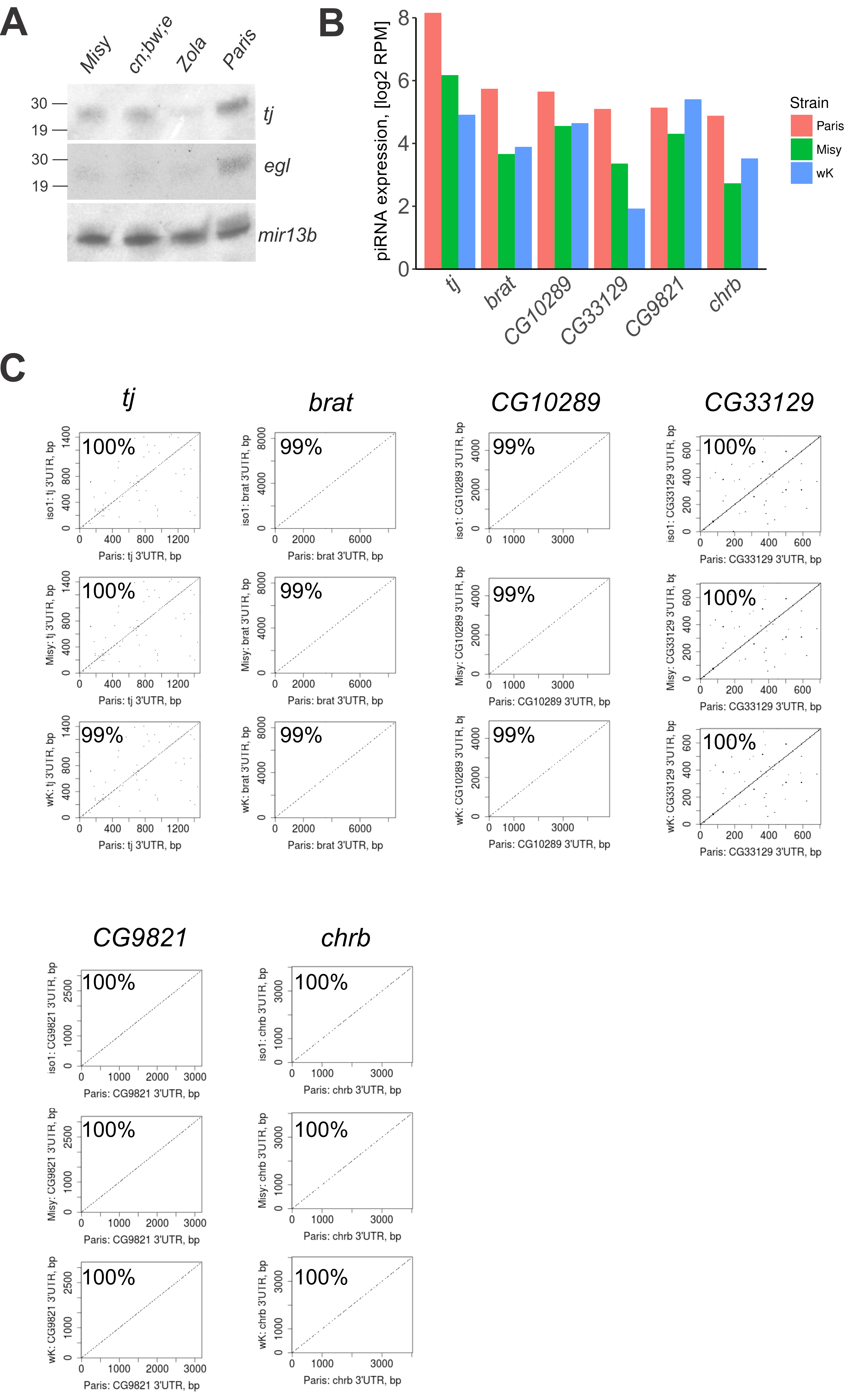

Supplement: S9 Fig — (A) Northern analysis of the small RNAs isolated from the ovaries of Misy, cn bw;e, Zola, Paris strains. Hybridization was done with the tj and egl antisense riboprobes. The lower panel represents hybridization to the mir-13b1 microRNA. (B) The number of 24–29 nt reads mapping to the six most productive genic piRNA clusters were normalized (RPM) and log2-transformed. (C) A comparison of genome sequences revealed no significant differences in the regions comprising six major genic piRNA clusters in Paris, Misy and wK strains. Dot plots show pairwise sequence alignments in iso-1, Paris, Misy, and wK strains; percent identity is indicated. (TIF) [file pgen.1006731.s010.tif]

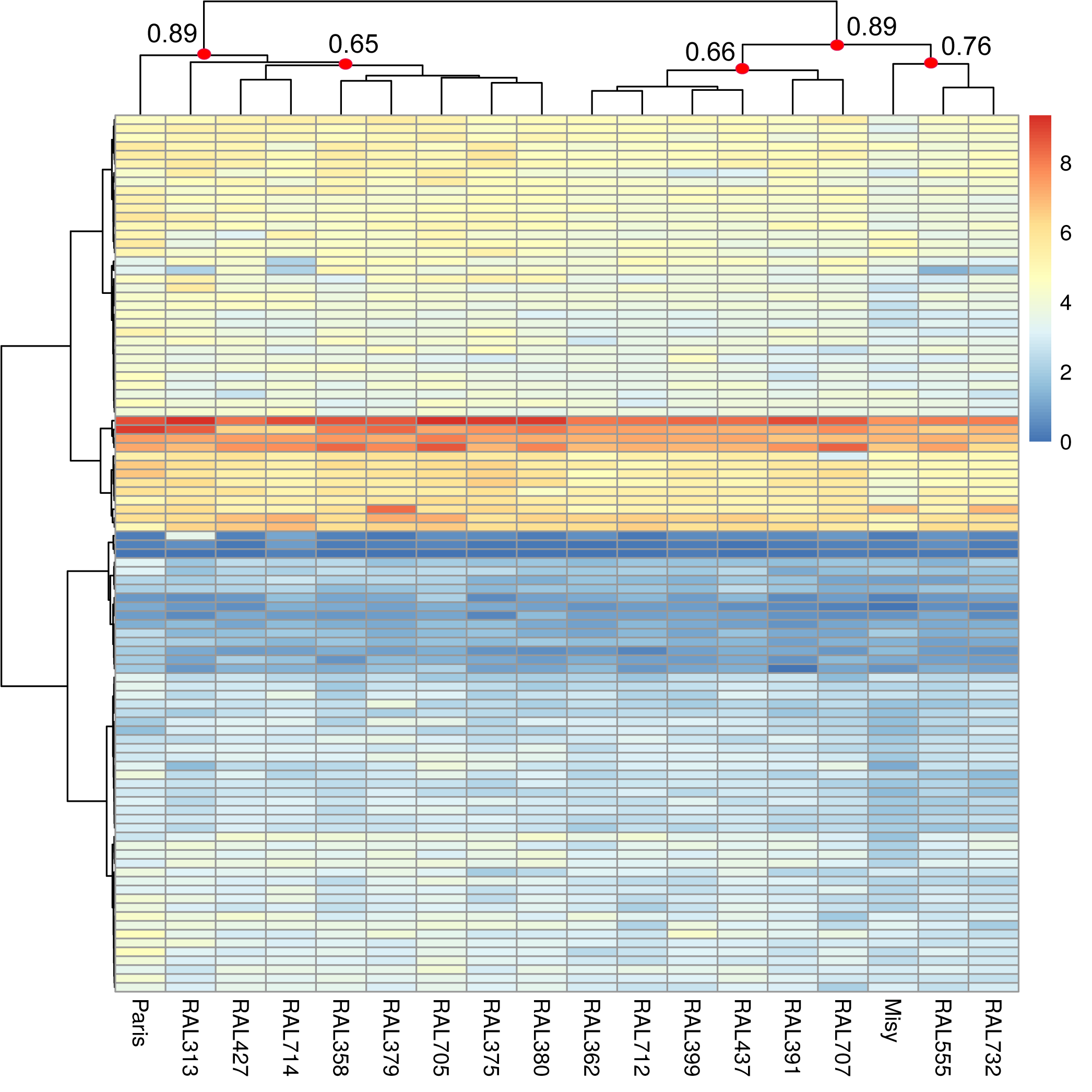

Supplement: S10 Fig — (TIF) [file pgen.1006731.s011.tif]

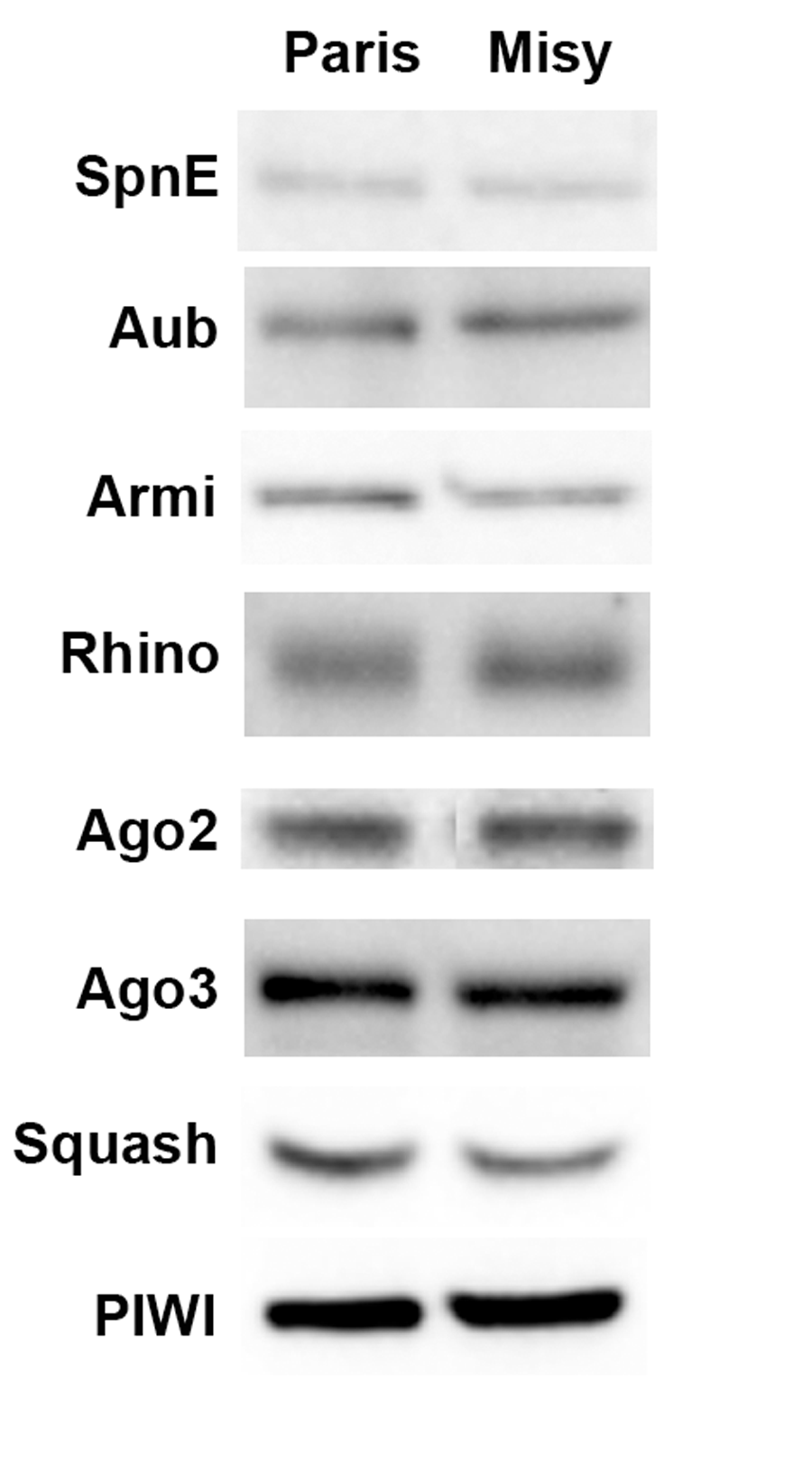

Supplement: S11 Fig — Antibodies used in the study are described in S1 File, Supporting Materials and Methods. (TIF) [file pgen.1006731.s012.tif]

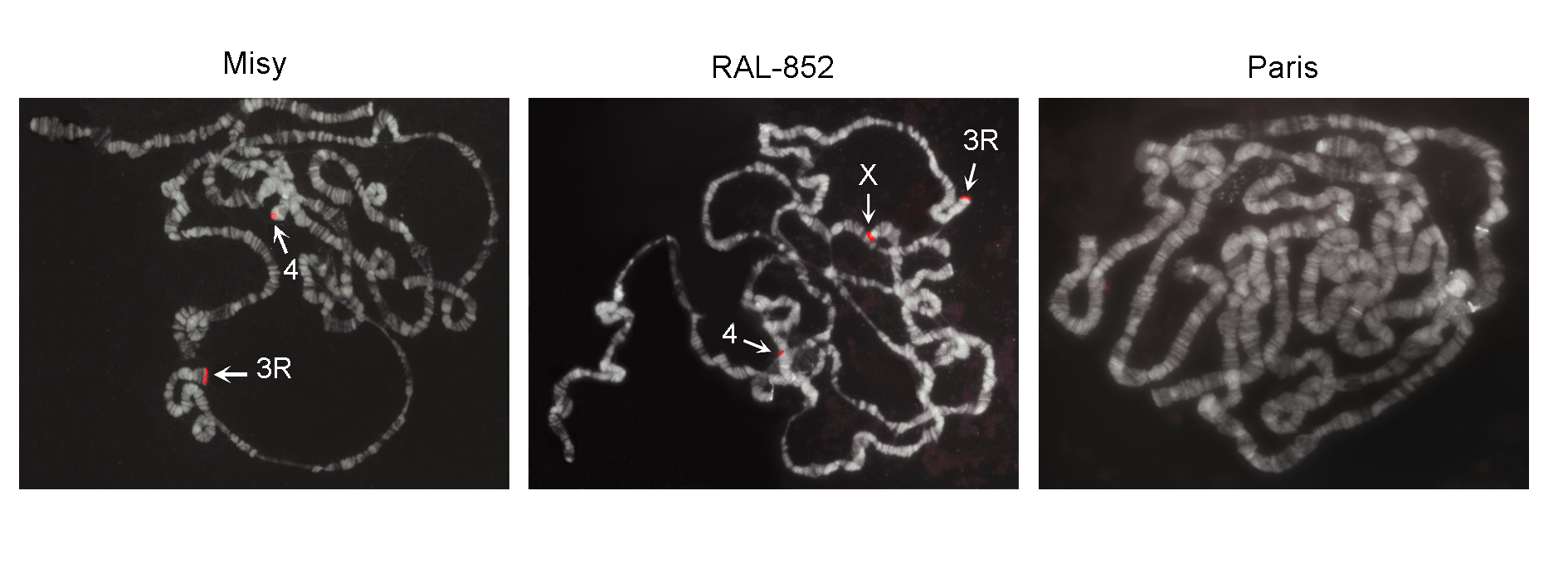

Supplement: S13 Fig — FISH analysis detected hybridization signals at some telomeres of Misy and RAL-852 strains. No hybridization signals were detected at the telomeres of the Paris strain. HeT-A is in red. Chromosomes are stained with DAPI (gray). (TIF) [file pgen.1006731.s014.tif]

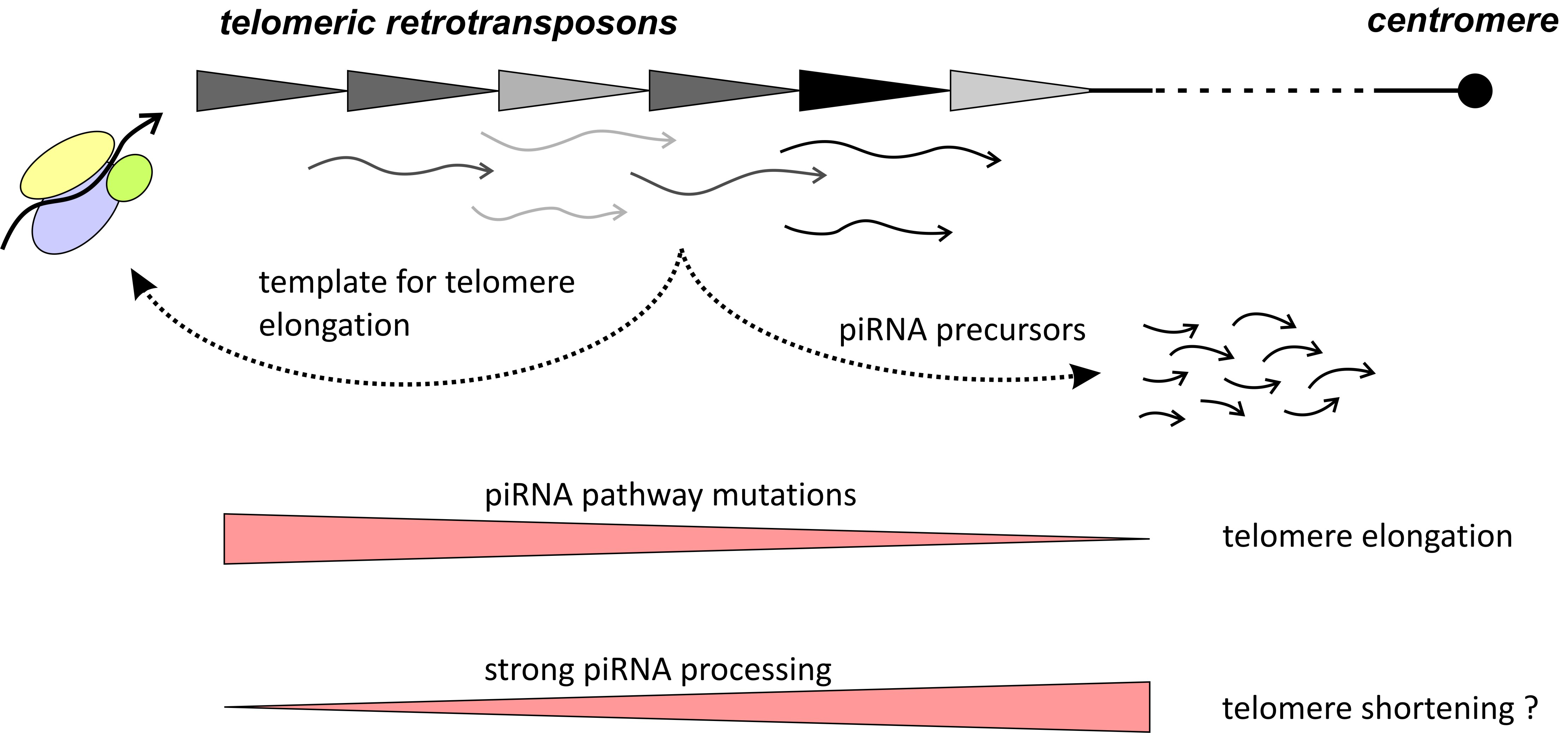

Supplement: S15 Fig — Telomeric repeats produce both piRNAs and their target mRNAs, which serve as templates for telomere elongation. piRNA elimination as a result of piRNA-pathway mutations causes telomere elongation [51]. An increased level of piRNA production as a result of the piRNA component polymorphism could cause an enhanced level of piRNAs specific to the main telomeric element HeT-A and telomere shortening. (TIF) [file pgen.1006731.s016.tif]
